# Assessing justice in California’s transition to electric vehicles

**DOI:** 10.1016/j.isci.2023.106856

**Published:** 2023-05-12

**Authors:** Eleanor M. Hennessy, Sita M. Syal

**Affiliations:** 1 Department of Energy Science and Engineering, Stanford University, Stanford, CA 94305, USA; 2 Department of Mechanical Engineering, University of Michigan, Ann Arbor, MI 48109, USA

**Keywords:** Energy resources, Energy policy, Energy management

## Abstract

Passenger vehicles are an essential form of transportation and contribute significantly to greenhouse gas emissions and criteria air pollution. The health and climate effects associated with their use disproportionately impact low-income communities and people of color. A shift from conventional vehicles to zero-emission vehicles is essential to meet climate targets and reduce inequities. The transition to clean transportation is an opportunity to uplift underserved and marginalized communities while building a sustainable transportation system. We assess justice in California’s transition to electric passenger vehicles by analyzing publicly available data on electric vehicle adoption and rebate use to measure justice in three areas: distribution of electric vehicles, allocation of state incentives, and the social and historical context of redlining. We find electric vehicle adoption and rebate use are lower in low-income and Latino-majority ZIP codes and in formerly redlined neighborhoods, indicating that California’s electric vehicle transition has not been just thus far.

## Highlights

• Formerly redlined neighborhoods receive fewer rebates and have fewer electric vehicles
• Latino-majority ZIP codes have the lowest electric vehicle penetration and rebates
• Racial disparities exist across all incomes for rebates and electric vehicle adoption
• Disparities in rebate allocation exceed disparities in electric vehicle penetration

## Graphical abstract

**Figure:**
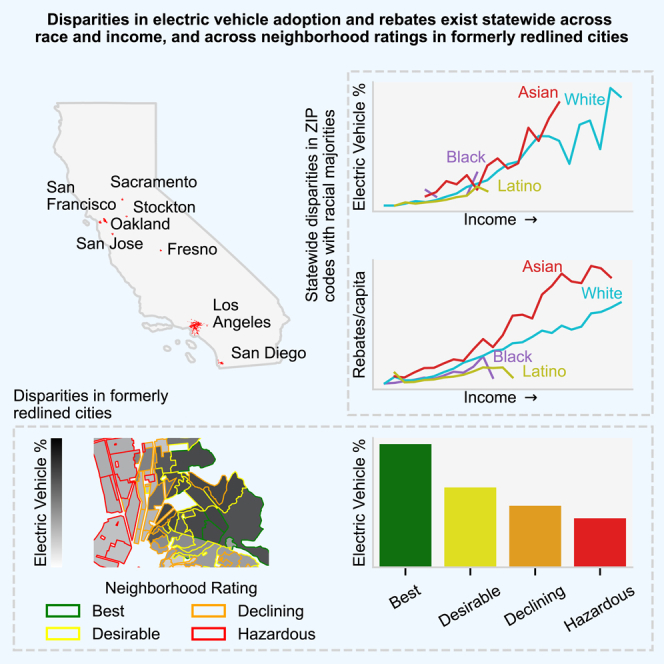

## Teaser

Energy policy; Energy justice

## Introduction

Climate change is one of the greatest challenges humanity faces. We are already seeing its impacts, and the Intergovernmental Panel on Climate Change (IPCC) predicts that without deep decarbonization, global temperature increase will reach 1.5 to 2°C by the end of the 21st century.^1^ The transportation sector is a key contributor to greenhouse gas (GHG) emissions. Transport is responsible for 15% of GHG emissions worldwide,^2^ 27% in the United States,^3^ and 42% in California.^4^ Within California, the focus of this work, light-duty (passenger) vehicles alone account for 28% of total GHG emissions.

Passenger vehicles are an essential form of transportation for many Californians. Public transit and other mobility services are feasible in some parts of the state and must be a part of the larger shift to clean mobility, but are not realistic mobility alternatives for much of the population. The convenience, opportunity, and agency associated with access to a personal vehicle are unmatched by other means of transportation.^5^ As of 2021, there were 29 million light-duty vehicles in use in California,^6^ serving a population of 39 million people.^7^ A transition to zero-emission vehicles (ZEVs) is an essential part of climate mitigation and the energy transition. Currently, the most promising zero-emission technologies for passenger vehicles are battery electric vehicles (BEVs), making up nearly two percent of California’s vehicle fleet,^6^ and fuel cell electric vehicles (FCEVs), making up less than one-10th of a percent of the fleet.^6^

California has ambitious climate goals and, as a national and global leader, is committed to accelerating the transition to ZEVs. The Advanced Clean Cars II regulation bans the sale of internal combustion engine (ICE) passenger vehicles and light trucks in the state beginning in 2035,^8^ and executive order B-55-18 sets a target of economy-wide carbon neutrality by 2045.^9^ While these policies will likely reduce GHG emissions, a focused effort is needed to ensure a just transition to ZEVs.

There have been numerous calls for a “just transition” to clean energy, of which transportation is an essential piece. These calls have come from governments,^10^^,^^11^ environmental justice groups,^12^^,^^13^ and scholars.^14^^,^^15^^,^^16^^,^^17^^,^^18^^,^^19^ Centering justice in the transition to clean energy and transportation systems requires consideration of access to clean transportation technologies, and the distribution of associated benefits and externalities.^20^ Energy justice, a subset of environmental justice, is often defined using three core tenets^14^: procedural justice, referring to the inclusion and participation of different groups in decision-making processes; distributive justice, referring to the distribution of benefits, both geographically and among different groups; and recognition justice, which refers to the valuation of certain groups of people compared to others. More recently, a fourth tenet, restorative justice, has been included in the definition, referring to the recognition of past injustices and action taken to right past wrongs.^19^ This is a forward-looking tenet to promote justice in practice.^21^ Similar concepts of justice are found in the transportation literature, which emphasizes understanding who is disadvantaged and who has access to opportunities related to the transportation system.^17^

Pursuing justice in the transition to clean transportation requires assessing the equity implications of components of the current system and potential solutions. Equity refers to the fair distribution of burdens and benefits, as well as the agency of individuals to meet their own needs.^16^^,^^22^ In this paper, we assess equity in sub-systems of California’s transportation system in order to assess justice in the state’s transition to clean passenger vehicles.

The current transportation system in the United States is inequitable.^23^ Along with GHGs, conventional ICE vehicles emit local pollutants that contribute to the formation of ground-level ozone and fine particulate matter, causing serious health impacts. Research has shown low-income and marginalized communities are exposed to more pollution than other groups from vehicle emissions and other sources.^24^^,^^25^ The same populations are more likely to live near major roadways, exposing them to more noise, pollution, and traffic.^26^ These disparities are tied to inequitable laws and policies. One key example is redlining, a racist lending practice with far-reaching impacts. Beginning in the 1930s in cities across the country, the Home Owners’ Loan Corporation (HOLC) created maps characterizing neighborhoods to determine eligibility for home loans. Neighborhoods were categorized as “A: best”, “B: still desirable”, “C: definitely declining”, and “D: hazardous.” These ratings were based, in part, on racial composition. Many people of color were ineligible^27^ and were denied the benefits of home ownership. The disparity in investment left a legacy of social and environmental impacts. While the 1968 Fair Housing Act officially ended redlining, the negative impacts on poorly rated neighborhoods and the marginalized communities living in them are still felt today. Present-day air quality is worse,^28^ surface temperatures are higher,^29^ there are more heat-related emergency room visits,^30^ and environmental impacts of transportation are higher^31^ in formerly redlined neighborhoods with low HOLC scores. Through the lens of restorative justice, these communities that have suffered disproportionate environmental, health, and social harms from past policies and practices should be prioritized in the transition to clean energy to make up for past injustices.

In this work, we explore the connection between present-day disparities in access to clean transportation and disinvestment in redlined neighborhoods to provide a basis for policy action to promote restorative justice in California’s transition to ZEVs. The central question in this work is the following: has the transition to ZEVs in California been just thus far? We perform three analyses to evaluate equity in three areas and help assess overall justice in the transition to ZEVs: a) participation: what is the current distribution of battery electric vehicles? b) benefits: who is benefiting from state government incentives? and c) social and historical context: how do disparities in access to BEVs and rebates relate to disinvestment in formerly redlined neighborhoods? The first two analyses are an assessment of distributive justice in the present day, while the third is an assessment of recognition justice, and provides a basis for pursuing restorative justice.

To assess participation, we use vehicle registration data and demographic data to identify which groups are over- and under-represented in BEV ownership. To understand the distribution of benefits, we look at recipients of BEV rebates in the Clean Vehicle Rebate Project (CVRP), the largest statewide ZEV rebate program in California.^32^ We rely on literature to determine which groups suffer from the burdens associated with conventional transportation (air pollution, traffic, and noise). Putting these metrics in the broader social context, we use data on historically redlined cities in California to assess how areas that have suffered from structural racism and disinvestment are faring in the current BEV transition. These analyses are conducted at the granular ZIP code level (as defined by the US Census). In a just transition, areas that have seen systemic disinvestment would be prioritized for investment in the energy transition through rebates and other programs.

We build on the work of past studies that have assessed the distribution of BEV rebates^33^^,^^34^^,^^35^ and equity in BEV adoption.^36^^,^^37^^,^^38^ While these studies have investigated various aspects of equity in BEV access, they are firmly rooted in the present day. In our assessment, we seek to link the current inequities in the transition to past injustices in the form of redlining. This work represents a first step toward restorative justice by measuring current distributive justice in communities impacted by past injustices.

Our assessment of the electric vehicle transition in California is timely. At the state level, the Advanced Clean Cars II regulation requires rapid adoption of BEVs, indicating the time for a just transition is now. At the federal level, the Inflation Reduction Act, which includes provisions for BEV subsidies, will increase BEV penetration nationwide. The transition to clean transportation is an opportunity to pursue restorative justice by prioritizing communities who have suffered from the current transportation system and who have been impacted by discriminatory policies and practices in the past. As California has led other states in BEV adoption, its trajectory may serve as an example; therefore, it is essential that the transition in California is just.

## Results and discussion

### Distributive justice in BEV adoption

**Figure 1:**
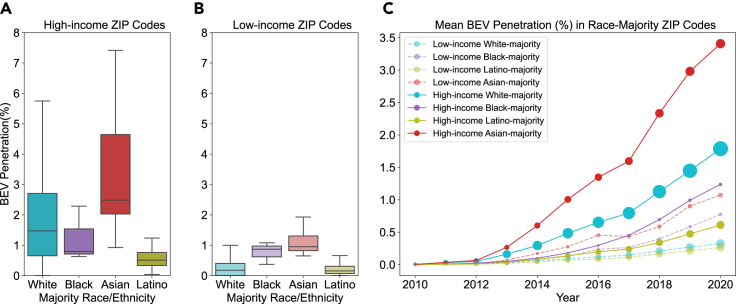
BEV penetration in high- and low-income ZIP codes with racial and ethnic majorities (A) BEV penetration in 2020 in high-income racial and ethnic-majority ZIP codes. Statistical significance of the difference between medians is as follows: White vs. Black: ∗∗∗; White vs. Asian: ∗∗∗∗; White vs. Latino: ∗∗∗∗; Black vs. Asian: ∗∗∗∗; Black vs. Latino: ∗∗; Asian vs. Latino: ∗∗∗∗. ns = no statistical significance, ∗ = p ≤ 0.05, ∗∗ = p ≤ 0.01, ∗∗∗ = p ≤ 0.001, ∗∗∗∗ = p ≤ 0.0001. The midline indicates the median, the upper and lower edges of the box indicate the upper and lower quartiles, and the whiskers indicate the minimum and maximum values excluding outliers. (B) BEV penetration in 2020 in low-income racial and ethnic-majority ZIP codes. Statistical significance of the difference between medians is as follows: White vs. Black: ∗; White vs. Asian: ∗∗∗∗; White vs. Latino: ∗∗∗∗; Black vs. Asian: ∗∗∗∗; Black vs. Latino: ∗∗∗; Asian vs. Latino: ∗∗∗∗. ns = no statistical significance, ∗ = p ≤ 0.05, ∗∗ = p ≤ 0.01, ∗∗∗ = p ≤ 0.001, ∗∗∗∗ = p ≤ 0.0001. The midline indicates the median, the upper and lower edges of the box indicate the upper and lower quartiles, and the whiskers indicate the minimum and maximum values excluding outliers. (C) Mean BEV penetration in ZIP codes in each racial/ethnic and income category from 2010 to 2020.

BEV penetration is a measure of current participation in the transition to electric vehicles. We define equitable BEV penetration as greater BEV penetration in low-income, Latino, and Black communities, who have been underserved by California’s current transportation system. We assess the distribution of BEV penetration in low- and high-income ZIP codes with different racial and ethnic majorities. Figures 1A and 1B show the distribution of BEV penetration in high- and low-income race-majority ZIP codes in 2020. Overall, BEV adoption rates are quite low. Median BEV penetration is below 3% for all race and income groupings. For all races and ethnicities, with the exception of Black-majority ZIP codes, high-income ZIP codes have higher median BEV penetration than low-income ZIP codes, suggesting that as seen in previous literature, BEV adoption is more prevalent in high-income areas. For Black-majority ZIP codes, the median BEV penetration is slightly higher in low-income ZIP codes. The sample size for Black-majority ZIP codes is very small (n = 6), and this finding is highly uncertain. All six Black-majority ZIP codes are located in the Los Angeles area, which overall has high levels of BEV penetration in the state. For both income groups, Asian-majority ZIP codes have the highest BEV penetration, and Latino-majority ZIP codes have the lowest. While the variation between races is small for low-income ZIP codes, it is larger for high-income ZIP codes. Our results align with other work in this area, which suggests low-income households and disadvantaged communities are less likely to have electric vehicles.^36^^,^^37^^,^^38^ Possible explanations for these disparities include language barriers, access to charging, availability of specific vehicles, cultural preferences, and structural inequalities.

There are variations in income level and the size of the racial majority in the selected categories, as shown in Figures S1 and S2. White-majority ZIP codes have larger racial majorities than other groups, with a median population percentage of 82.4% in high-income White-majority ZIP codes, and 87.3% in low-income White-majority ZIP codes. Black-majority ZIP codes have the next largest median population percentage in high-income ZIP codes (69.8% in Black-majority ZIP codes), but have the smallest majority in low-income ZIP codes (60.9%). Latinos have the next highest majority in low-income ZIP codes (70.4%), but the second lowest majority in high-income ZIP codes (62.6%). Asian-majority ZIP codes have fairly small majorities (59.0% in high-income ZIP codes, and 61.2% in low-income ZIP codes). These discrepancies suggest that White-majority ZIP codes may be more representative of the White individuals living in them than other racial-majority neighborhoods due to their higher population percentages. Low-income Latino-majority ZIP codes may be more representative than high-income Latino-majority ZIP codes, and high-income Black-majority ZIP codes may be more representative than low-income Black-majority ZIP codes. There are also variations in income distribution within each racial and income category, as shown in Figure S2. Within high-income ZIP codes, Asian-majority ZIP codes have the highest median income ($113,577), followed by White-majority ($94,863), Black-majority ($89,364), and Latino-majority ($71,542). In low-income ZIP codes, Asian-majority ZIP codes again have the highest median income ($59,217), followed by Black-majority ($52,605), Latino-majority ($46,580), and White-majority ZIP codes ($45,784). As shown in Figure S3, low-income White- and Latino-majority ZIP codes are concentrated in more rural parts of the state (Northern California for White-majority and the Central Valley for Latino-majority), than the Black-majority and Asian-majority ZIP codes that are concentrated in more urban areas, which may contribute to the disparities seen in low-income ZIP codes.

In Figure 1C, we track how mean BEV penetration has changed over time. While programs have been implemented in the state to improve access to BEVs in lower-income and non-White communities, the data suggest that the disparities have not significantly decreased. BEV adoption in high-income Asian-majority ZIP codes has consistently outpaced adoption in all other race-majority ZIP codes, and the adoption rate in this subset appears to be increasing faster than in the others. Low-income Latino-majority ZIP codes have seen the slowest growth and the lowest BEV penetration since the introduction of BEVs to the market. In high-income Black-majority ZIP codes, adoption was initially very low, but has been increasing rapidly.

There is evidence that exposure to BEV technology leads to higher adoption rates and clustering of BEV adoption. This “neighborhood effect” has been addressed in the literature.^39^^,^^40^ To confirm this pattern in our data, we compare BEV penetration in each ZIP code to the average BEV penetration in neighboring ZIP codes (see Figure S6). Across all races, and in both low-income and high-income ZIP codes, there is strong correlation between BEV penetration in a ZIP code and the BEV penetration in neighboring ZIP codes. Latino-majority ZIP codes tend to have somewhat lower BEV penetration than neighboring ZIP codes. In high-income ZIP codes, Asian-majority ZIP codes tend to have lower BEV penetration than neighboring ZIP codes, while the reverse is true for low-income Asian-majority ZIP codes. Low-income ZIP codes tend to have lower BEV penetration than their neighbors, while high-income ZIP codes tend to have higher BEV penetration than their neighbors.

Without individual-level data, it is unclear if the BEVs belong to individuals of the racial majority in each ZIP code. Given this uncertainty, we also compute the population-weighted average BEV percentage by race in all high- and low-income ZIP codes, assuming that BEVs are distributed proportionately to the racial makeup of the population (see Table S1). The results of this secondary analysis are consistent with our analysis using racial and ethnic majorities, showing that Asian populations have the highest BEV penetration and Latino populations have the lowest in both high- and low-income ZIP codes. In high-income ZIP codes, Black populations have the second lowest BEV penetration, while in low-income ZIP codes White populations have the second lowest. We also test the impact of using a county-specific income cutoff from the California Department of Housing and Community Development^41^ to define low income, as shown in Figure S16, which has little impact on the results.

### Equitable access to the benefits of BEV rebates

Subsidies and incentive programs have been a key strategy for encouraging the transition to electric vehicles, and represent a financial benefit delivered by the state separate from the direct benefits of BEV ownership. Equitable distribution of benefits is an important component of a just transition. This means more rebates would be directed to low-income, Latino, and Black communities, who have largely borne the burden of California’s current transportation system.^25^

We analyze the number of rebates per capita awarded through CVRP as a measure of the equity of incentive benefits. CVRP began in 2010 and has operated continuously through the present. It is the largest rebate program in the state, having given out more than 500,000 rebates, totaling more than $1 billion in funding. The program’s stated goal is “to promote the production and use of ZEVs”.^32^ While the program was not initially intended to promote equity, eligibility was limited to customers below a specified income cap beginning in March 2016 (and adjusted in November 2016). Additionally, an increased rebate amount is available for households whose income is less than 400% of the federal poverty limit.^32^The current income cap of $200,000 for joint-filers is well above typical cutoffs used to define low income in the state, which range from $51,850 to $129,150 depending on the county.^41^

We choose to focus on CVRP as it is the largest incentive program in the state and is available statewide. Other rebate programs in the state are much smaller in scale. Clean Cars for All has provided less than $200 million in incentives, and the Clean Vehicle Assistance Program has provided roughly $60 million in incentives.^42^ While these programs target low-income and disadvantaged communities, they do not have the funds to provide as much assistance as CVRP. Additionally, Clean Cars for All, up to this point, has not been a statewide program, and has been implemented only in select Air Districts. Additional incentive programs, such as the federal tax credit and programs at the municipality level are outside of the scope of this work and have been discussed by others.^43^^,^^44^^,^^45^

**Figure 2:**
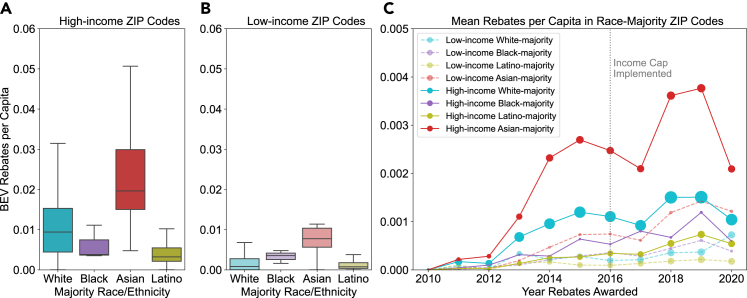
BEV rebates per capita in high- and low-income ZIP codes with racial and ethnic majorities (A) Cumulative BEV rebates per capita in high-income racial and ethnic-majority ZIP codes. Statistical significance of the difference between medians is as follows: White vs. Black: ∗∗∗; White vs. Asian: ∗∗∗∗; White vs. Latino: ∗∗∗∗; Black vs. Asian: ∗∗∗∗; Black vs. Latino: ∗∗; Asian vs. Latino: ∗∗∗∗. ns = no statistical significance, ∗ = p ≤ 0.05, ∗∗ = p ≤ 0.01, ∗∗∗ = p ≤ 0.001, ∗∗∗∗ = p ≤ 0.0001. The midline indicates the median, the upper and lower edges of the box indicate the upper and lower quartiles, and the whiskers indicate the minimum and maximum values excluding outliers. (B) Cumulative BEV rebates per capita in low-income racial and ethnic-majority ZIP codes. Statistical significance of the difference between medians is as follows: White vs. Black: ∗; White vs. Asian: ∗∗∗∗; White vs. Latino: ∗∗∗∗; Black vs. Asian: ∗∗∗∗; Black vs. Latino: ∗∗∗; Asian vs. Latino: ∗∗∗∗. ns = no statistical significance, ∗ = p ≤ 0.05, ∗∗ = p ≤ 0.01, ∗∗∗ = p ≤ 0.001, ∗∗∗∗ = p ≤ 0.0001. The midline indicates the median, the upper and lower edges of the box indicate the upper and lower quartiles, and the whiskers indicate the minimum and maximum values excluding outliers. (C) Mean annual BEV rebates per capita from 2010 to 2020.

Figures 2A and 2B show the distribution of rebates per capita in high- and low-income racial/ethnic-majority ZIP codes. As with BEV penetration, we find Asian-majority ZIP codes receive the highest number of rebates per capita, followed by White-majority ZIP codes. Black- and Latino-majority ZIP codes receive fewer rebates. Again, we note that while the racial disparities are relatively small for low-income ZIP codes, they are quite large for high-income ZIP codes. This suggests a low income level may be restrictive for all races and ethnicities, but even when income is sufficiently high for BEV purchase to be possible, some races are more likely to receive rebates. Given that CVRP uses rebates, rather than point-of-sale discounts, it is likely not sufficient for those who lack the necessary capital to pay the upfront cost. Additionally, CVRP does not assist with financing, which is another important factor. There is growing evidence that people of color rely more heavily on financing for vehicle purchases and pay higher premiums than White populations,^46^ which may play a role in the disparities seen in both rebate allocation and BEV adoption.

As with BEV penetration, we perform a secondary analysis of BEV rebate allocation, computing the population-weighted mean BEVs per capita by race in high- and low-income ZIP codes, assuming rebates are allocated proportionately to the population (see Table S2). In this analysis, we find that for both low-income and high-income ZIP codes, Asian populations receive the most rebates per capita, and Latino populations receive the fewest, which is aligned with our findings from the racial-majority analysis. Similarly, using a county-specific income cutoff has little impact on the results (see Figure S17). Our findings are consistent with the results of other studies, which suggest BEV rebates are used more frequently by White, wealthy households.^47^^,^^48^ As with BEV penetration, we see that there is a strong correlation between rebates per capita in a given ZIP code and the rebates per capita in neighboring ZIP codes, suggesting the neighborhood effect is present in rebate allocation, as well as in BEV adoption (see Figure S7). As with BEV penetration, low-income ZIP codes tend to have lower rebates per capita than their neighbors, while the reverse is true for high-income ZIP codes.

Another factor that could contribute to the disparities in rebate allocation is differences in personal vehicle ownership. Some groups may rely more on public transit or shared mobility than others. To assess personal vehicle ownership, we compare the median vehicles per capita in each race and income category, shown in Figure S4. Vehicle ownership is similar in high-income and low-income ZIP codes, while there is more variability in low-income ZIP codes. For both low- and high-income ZIP codes, White-majority ZIP codes have the highest number of vehicles per capita. In high-income ZIP codes, Asian-majority ZIP codes have the lowest vehicles per capita. This suggests that BEV rebate allocation cannot be fully explained by personal vehicle ownership, as Asian-majority ZIP codes receive the most rebates per capita.

Figure 2C shows how the number of rebates per capita in ZIP codes in each demographic category have changed since CVRP began. As with BEV penetration, high-income Asian-majority ZIP codes have consistently received the highest number of rebates per capita. A 2016 income cap restricted rebates to households making under $200,000 per year. While this likely explains the dip in total rebates per capita the following year, it does not appear to have had a large impact on the disparity in rebates awarded. However, the income cap is much higher than our cutoff for low-income ZIP codes, and households in both income categories are eligible for the rebate. Likewise, the income cutoff for increased rebate amounts is higher than our cutoff for low income, and as a result, we would expect to see any effects in both income categories. Other studies suggest that the income cap did somewhat improve distributional equity^48^ and that after the income cap, rebates increased in low-income areas located near higher-income areas already receiving a high number of rebates.^34^

In 2020, the total number of rebates per capita again decreased, due to the COVID-19 pandemic. According to a report by the Center for Sustainable Energy, the statewide administrator of CVRP, rebate applications decreased by 43% in 2020 due to the pandemic.^49^ The demographics of rebate applicants during this time period also shifted, with the share of female applicants increasing, the share of Asian applicants decreasing, and the share of Latino applicants increasing.^49^ Overall vehicle sales in California decreased by 22% in 2020 compared to the previous year, while the share of new BEV registrations increased from 5.1% to 6.2%.^50^ Total vehicle sales in 2021 and 2022 have remained below pre-COVID-19 levels, while the share of new BEV registrations increased to 17.1% in 2021. As the market continues to recover, future analysis could investigate the changes in the distribution of BEV rebates post-pandemic.

### Understanding social and historical context through redlining

**Figure 3:**
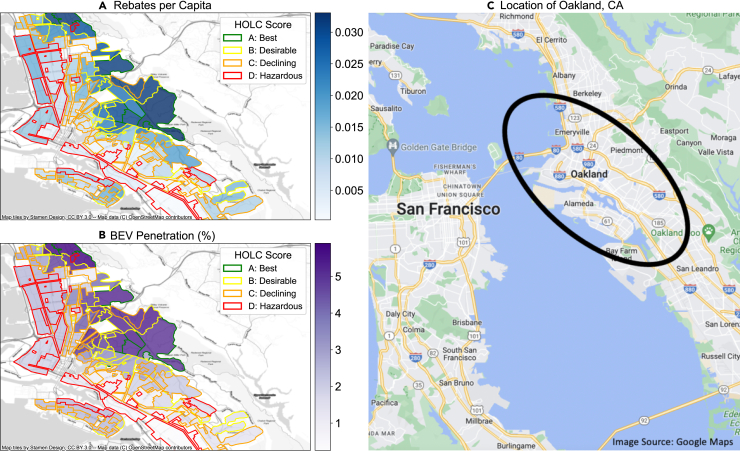
BEV penetration and rebates per capita in formerly redlined neighborhoods in Oakland, California (A) Cumulative rebates per capita awarded in neighborhoods in Oakland, California. Darker blue indicates higher rebates per capita. The color of the outlines corresponds to the HOLC score in the neighborhood, with green corresponding to an “A” grade, yellow corresponding to a “B” grade, orange to a “C” grade, and red to a “D” grade. (B) BEV penetration in 2020 in neighborhoods in Oakland, California. Darker purple indicates higher BEV penetration. The color of the outlines corresponds to the HOLC score in the neighborhood, with green corresponding to an “A” grade, yellow corresponding to a “B” grade, orange to a “C” grade, and red to a “D” grade. (C) Location of Oakland, California. Figures showing additional cities can be found in the SI (Figures S8–S14).

While the distribution of BEVs and allocation of rebates provide a baseline understanding of equity in the transition, understanding social and historical context, in the form of systemic and place-based inequities, is necessary to assess justice. People’s abilities to participate in the transition are impacted by the additional burdens they face, and a just transition requires prioritizing communities that have faced systemic challenges or need more assistance. To understand how communities burdened by past policies and disinvestment are faring in the current transition, we relate the previous two metrics (BEV penetration and rebates per capita) to HOLC scores, used to determine eligibility for loans in redlined cities. We conduct this analysis for all eight cities in California where redlining took place. As an example, Figure 3 shows rebates per capita and BEV penetration in Oakland, California. Figures showing additional cities can be found in the SI (Figures S8–S14). In general, the highest BEV penetration (dark purple areas in the map), and highest rebates per capita (dark blue areas on the map) occur in neighborhoods with “A” and “B” grades, outlined by green and yellow in the map. This pattern is strongest in Oakland, Los Angeles, and San Diego.

**Figure 4:**
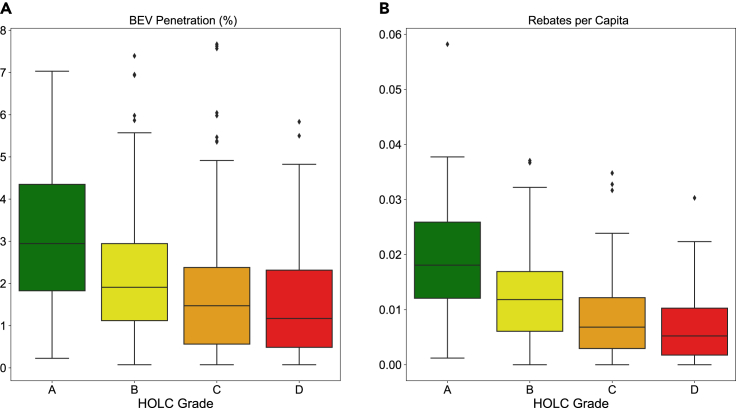
BEV penetration and rebates per capita in formerly redlined cities in California (A) BEV Penetration in redlined neighborhoods. Statistical significance of the difference between medians is as follows: A vs. B: ∗∗∗∗; A vs. C: ∗∗∗∗; A vs. D: ∗∗∗∗; B vs. C: ∗∗∗∗; C vs. D: ns. ns = no statistical significance, ∗ = p ≤ 0.05, ∗∗ = p ≤ 0.01, ∗∗∗ = p ≤ 0.001, ∗∗∗∗ = p ≤ 0.0001. The midline indicates the median, the upper and lower edges of the box indicate the upper and lower quartiles, and the whiskers indicate the minimum and maximum values excluding outliers. (B) Rebates per capita in redlined neighborhoods. Statistical significance of the difference between medians is as follows: A vs. B: ∗∗∗∗; A vs. C: ∗∗∗∗; A vs. D: ∗∗∗∗; B vs. C: ∗∗∗∗; B vs. D: ∗∗∗∗; C vs. D: ∗. ns = no statistical significance, ∗ = p ≤ 0.05, ∗∗ = p ≤ 0.01, ∗∗∗ = p ≤ 0.001, ∗∗∗∗ = p ≤ 0.0001. The midline indicates the median, the upper and lower edges of the box indicate the upper and lower quartiles, and the whiskers indicate the minimum and maximum values excluding outliers.

Figure 4 shows the distribution of BEV penetration and rebates per capita across all eight cities in California that underwent redlining. Median BEV penetration in the “Best” (A grade) neighborhoods is approximately three times the penetration in “Hazardous” (D grade) neighborhoods. This suggests areas that have suffered from systematic disinvestment are now seeing lower access to BEVs. The disparity in rebates per capita is even greater, with rebates per capita in “Best” neighborhoods approximately five times higher than rebates per capita in “Hazardous” neighborhoods. This suggests disinvestment in the form of government subsidies is continuing in these areas. These communities have already suffered from a lack of investment in housing and have missed out on associated benefits. Through the lens of restorative justice, these communities should be prioritized for new government spending to correct for past disinvestment.

### Racial and ethnic disparities across all income levels

Results shown in the previous sections suggest that race, income, and social and historical context are important factors in BEV adoption and BEV incentives. Racial differences in adoption and rebates exist in both the high- and low-income groups, though the differences are far greater in the high-income group. One explanation for the smaller differences in the low-income group is that BEV adoption may be suppressed due to an inability to participate caused by insufficient income. BEVs are more expensive than ICE vehicles, and rebate amounts may not be sufficient to overcome the price difference for low-income households. In addition to income, credit score and access to loans are important, as well as generational wealth that may not be reflected by income. There is also a question of the impact of the distribution of incomes within the high- and low-income groupings for each race. For example, Black-majority high-income ZIP codes have, on average, lower median incomes than White-majority high-income ZIP codes as shown in Figure S2.

**Figure 5:**
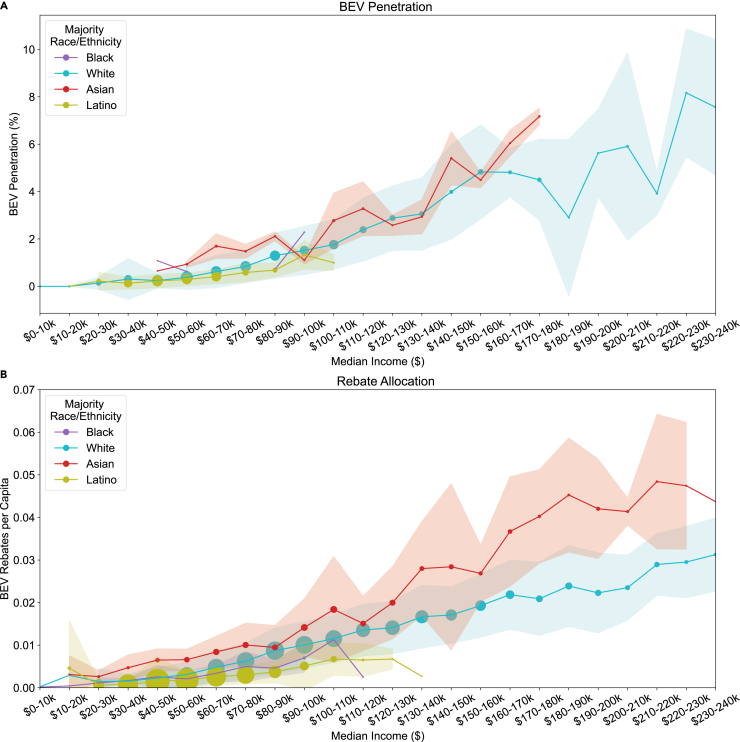
BEV penetration and BEV rebates per capita in ZIP codes with racial and ethnic majorities across income levels (A) BEV penetration in racial and ethnic-majority ZIP codes by median income. Marker size corresponds to the number of ZIP codes, marker location indicates the mean BEV penetration, and shading indicates the standard deviation of BEV penetration in each income bracket and racial/ethnic-majority. (B) BEV rebates per capita in racial and ethnic-majority census tracts. Note that census tracts are used in this analysis due to their finer granularity and larger sample size. Marker size corresponds to the number of census tracts, marker location indicates the mean rebates per capita, and shading indicates the standard deviation of rebates per capita in each income bracket and racial/ethnic-majority.

We further explore the relationship between race and income as they relate to participation and benefits in Figures 5A and 5B, which show mean BEV penetration and rebates per capita in race-majority ZIP codes and census tracts with median incomes across a range of income brackets. In this analysis we use rebate data at the census tract level due to the finer granularity and larger number of census tracts. It is apparent that racial and ethnic disparities persist at all income levels and increase at higher income levels. This suggests a reduction in the price of BEVs alone will not be sufficient to achieve equity in the electric vehicle transition. We focus on race and income, but other factors such as access to charging infrastructure and cultural values may play a role.^51^

### Interactions between rebate allocation and BEV distribution

Throughout our analysis, we have primarily treated BEV penetration as separate from rebate allocation. However, it is likely that rebate allocation has influenced the distribution of BEV adoption, and vice versa. To assess if the distribution of rebates matches the distribution of BEVs, we compute the cumulative BEV rebates between 2010 and 2020 per registered BEV in 2020 in ZIP codes in each race and income category, as shown in Figure S5. While it is likely that not all vehicles have remained registered in the same ZIP code in which they were located when a rebate was originally received, this metric demonstrates the relationship between rebate allocation and BEV allocation. Using this metric, Asian-majority ZIP codes still receive the most rebates in both high- and low-income ZIP codes. Latino-majority ZIP codes receive more rebates by this metric and are the group with the second-most rebates received per BEVs registered for both high- and low-income ZIP codes. Median rebates per registrations are similar between low-income and high-income ZIP codes.

We also compare disparities in BEV penetration (Figure 1) to disparities in rebate allocation (Figure 2). We see greater disparities in rebate allocation than in BEV penetration. In high-income ZIP codes, Asian-majority ZIP codes receive roughly twice as many rebates per capita as White-majority ZIP codes, five times as many as Black-majority ZIP codes, and six times as many as Latino-majority ZIP codes. In contrast, BEV penetration in Asian-majority ZIP codes is 1.7, 3.1, and 4.9 times larger than in White-majority, Black-majority, and Latino-majority ZIP codes, respectively. In low-income ZIP codes, the difference between disparities in BEV penetration vs. rebate allocation is even larger, with Asian-majority ZIP codes having BEV penetration 5.3, 1.1, and 6 times larger than White-majority, Black-majority, and Latino-majority ZIP codes, respectively, compared to 9.2, 2.2, and 10 times the rebate allocation. This suggests that disparities in rebate allocation cannot fully be explained by the distribution of BEVs.

Another logical hypothesis is that BEV distribution could be explained by rebate allocation. If this were the case, we would expect to see a change in disparities between low-income and high-income BEV adoption after CVRP implemented its income cap in mid-2016. While the BEV penetration rate increases more rapidly in low-income Black-majority ZIP codes and low-income Asian-majority ZIP codes after this point, there is also an increase in high-income ZIP codes of all majorities, as seen in Figure 1. However, as mentioned previously, the CVRP income cap is much higher than both our income cutoff and state-defined cutoffs for low-income characterization, which would make changes less visible. Future work could investigate this relationship more thoroughly.

### Additional considerations

We have focused on adoption of BEVs, but a similar analysis could assess other vehicle technologies as well. BEVs are currently the most prominent ZEV technology and are poised to be a key part of the energy transition. However, there are additional equity concerns with the technology, and our focus on BEVs should not be taken as an explicit endorsement of the technology. There are emissions and health concerns with battery manufacturing, primarily located in China.^52^ Extraction of lithium, nickel, cobalt, and other minerals in batteries also causes a range of environmental justice concerns. Cobalt primarily comes from the Democratic Republic of the Congo, where there are issues with child labor.^53^^,^^54^^,^^55^ Nickel mining is associated with pollution and environmental damage.^56^ Vast reserves of lithium are concentrated in South America, where water scarcity and indigenous lands pose challenges.^57^ The Inflation Reduction Act requires a shift to battery material sourcing and manufacturing in the United States,^58^ but many U.S. lithium deposits are on Tribal land.^59^ These concerns are not trivial. Further investigation into the environmental justice and equity implications of potential technologies is needed.

We have focused on California in this study given the availability of data and the state’s leadership in the transition. As electric vehicle penetration increases in other states it will be important to track justice metrics there as well. Future work could incorporate additional rebate programs, such as Clean Cars for All, or assess justice at the local level, as many municipalities have their own ZEV incentive programs. The approach we have taken could also be used in other areas of the clean energy transition (e.g., rooftop solar, behind-the-meter storage, or building energy efficiency measures).

In this work we do not distinguish between used and new electric vehicles in our analysis. While it is likely that used vehicles may have a significant role to play in making electric vehicles more accessible to lower income families,^60^ the lack of publicly available data on used vehicle sales makes analysis on this topic challenging at the current time. Additionally, while used vehicles are generally less expensive than new vehicles, they also tend to have shorter ranges due to battery degradation. This raises additional questions about relying on used electric vehicles to meet the passenger vehicle needs of lower-income individuals. As more data becomes available on used electric vehicles, future work could characterize these dynamics.

### Limitation of the study

This analysis is conducted at the ZIP code level due to a lack of publicly available individual-level data. While data privacy is important, the aggregation of rebate and vehicle ownership data to the ZIP code level may mask inequities. In this analysis, we are limited to analyzing participation by and benefits distributed to White, Black, Asian, and Latino populations as there are no ZIP codes or census tracts with a majority of Native Americans, Pacific Islanders, or other races. As a result, we are ignorant to inequities these communities face in the transition to clean transportation. Additionally, there are very few Black-majority ZIP codes, resulting in high uncertainty.

Without individual-level data, we cannot identify which members of each ZIP code and census tract actually own the BEVs or received the rebates. A rebate in a Black-majority census tract could have gone to a White individual, or vice versa. We have characterized each ZIP code based on median income, but there is no guarantee that BEVs present in a low-income ZIP code are not owned by individuals with incomes well above the median. More transparent data could improve our ability to track equity during the transition.

In addition to data on the allocation of BEVs and BEV rebates, data on decision-making processes around vehicle decarbonization are needed to be able to fully assess procedural justice in addition to distributive justice. The California Air Resources Board, who makes decisions about statewide rebate programs, has an advisory board for environmental justice,^61^ but there is a lack of participation or feedback built into the program. Future work could explore how community members from various backgrounds could be effectively included in decisions around vehicle incentives and vehicle decarbonization.

In this study, we have focused on race, ethnicity, and income level as key sociodemographic factors in understanding justice in the transition to clean transportation. While these are important factors for understanding equity and justice, additional factors that we have not accounted for here may impact the distribution of BEVs and BEV rebates we have shown. In particular, education level,^37^^,^^48^^,^^62^^,^^63^^,^^64^ home type and ownership,^48^^,^^62^ age,^62^^,^^64^^,^^65^ and gender^62^^,^^65^ have been shown to be important factors in BEV adoption and rebate distribution in a variety of locations. Additional factors, such as availability of public charging stations and commute distance may also have an impact.^66^ The social context of the area of study is an important consideration as well, and specific findings resulting from this study focused on California may not be generalizable to other regions.

### Conclusions

The goal of this work was to assess justice in the transition to electric vehicles in California in the context of past systemic injustices. Our results suggest that the current transition is not just, and that there is an opportunity to limit further injustices in the transition moving forward. We approached this problem by evaluating equity in current BEV adoption, rebate allocation, and social and historical context. To have a truly just transition, communities that have seen a lack of investment in the past should be prioritized for investment during the clean energy transition. We have demonstrated that there are disparities in both BEV penetration and rebate allocation, with high-income ZIP codes having both higher BEV penetration and a higher number of rebates per capita. In addition to income-related inequity, there is evidence of racial inequity, with Asian-majority and White-majority ZIP codes receiving more benefits than Black- and Latino-majority ZIP codes. There are also severe inequities in historically redlined cities, indicating that communities that have consistently borne the burden of the current transportation system and many other societal burdens are being left behind.

The clean energy transition is a significant opportunity to create a more just energy system, and to uplift underserved communities. Prioritizing low-income communities, communities of color, and communities living in areas facing chronic disinvestment would be a step toward justice. While the transition is not just in its current state, there is still time for this to be corrected. This will require more than just reducing the cost of BEVs. Increased incentives for used vehicles, point-of-sale rebates, and active engagement of underserved communities in planning and design processes, among other strategies, have been suggested as a start.^67^ Eliminating barriers in rebate program applications and reducing program timelines could also be beneficial.^68^ As a leader in the electric vehicle transition, California is in a unique position. The recent Advanced Clean Cars II legislation will accelerate the transition to electric vehicles. Already, other states are following California’s lead with New York proposing a similar mandate to require 100% ZEV sales by 2035.^69^ If California is successful in creating a just transition, other states will likely follow, and if it is unsuccessful, it will serve as a cautionary tale. Putting justice front and center in policies and plans for the energy transition will ensure that at the end of the day we are left not only with a low carbon future, but also a more sustainable one that is built for everyone.

## STAR★Methods

### Key resources table

**Table:** 

REAGENT or RESOURCE	SOURCE	IDENTIFIER
**Deposited data**		

Clean Vehicle Rebate Project (CVRP) electric vehicle rebate data	Center for Sustainable Energy	https://cleanvehiclerebate.org/en/rebate-statistics
Aggregated vehicle registration data	California Energy Commission	https://www.energy.ca.gov/files/zev-and-infrastructure-stats-data
Demographic data	American Community Survey, U.S. Census Bureau	https://www.census.gov/programs-surveys/acs/data.html
TIGER/Line shapefiles	U.S. Census Bureau	https://www.census.gov/geographies/mapping-files/time-series/geo/tiger-line-file.2019.html#list-tab-790442341
Historically redlined cities shapefiles	Mapping Inequality, University of Richmond	https://dsl.richmond.edu/panorama/redlining/#loc=5/39.096/-94.592

**Software and algorithms**		

Python 3.9.16	Python Software Foundation	https://www.python.org/downloads/release/python-3916/
Analysis code	Authors	https://doi.org/10.5281/zenodo.7865744

### Resource availability

#### Lead contact

Further information and requests for resources should be directed to and will be fulfilled by the lead contact, Eleanor M. Hennessy (emh@stanford.edu)

#### Materials availability

This study did not generate any new materials.

### Method details

#### Data sources and approach

We use the following publicly available data sources.

• Hybrid and electric vehicle rebate data from the Clean Vehicle Rebate Project (CVRP), hosted by the Center for Sustainable Energy.^70^ This dataset includes all rebates given between 2010 and 2020 through the program, and contains information about the type of vehicle purchased, rebate amount, and census tract and ZIP code in which the recipients live.
• Aggregated vehicle registration data from the Department of Motor Vehicles hosted by the California Energy Commission.^6^ This dataset provides the number of light-duty vehicles by fuel type registered in each ZIP code in California on an annual basis from 2010 to 2020.
• Demographic data from the U.S. Census American Community Survey.^71^ This dataset contains ZIP code- and census tract-level data on race, ethnicity, and household income. We use 5-year estimates from 2019.
• TIGER/Line maps from the U.S. Census Bureau for the ZCTA and Census Tract level.^72^ ZCTAs are matched to U.S. ZIP codes to allow for analysis.
• Historical redlining maps from the Mapping Inequality project at the University of Richmond.^73^ This dataset contains shapefiles of cities that underwent redlining with the HOLC score for each neighborhood.

We conduct three separate analyses to assess justice in each portion of our framework. First, we look at trends in BEV ownership by race, ethnicity, and income at the ZIP code-level in California to evaluate distributive justice in participation in the electric vehicle transition. We include ZIP codes with a majority of White non-Latinos (“White”), Black non-Latinos (“Black”), Asian non-Latinos (“Asian”), and Hispanic/Latinos (“Latino”). The term “Hispanic” is a language-based term and generally refers to individuals with roots in Latin America or Spain, while “Latino” is generally used to refer to individuals whose origin is Latin America. As the U.S. Census groups these two together in the “Hispanic or Latino” category, we treat them as one group in our analysis, but refer to them as “Latino” for brevity. Second, we use data from California’s CVRP to assess the distribution of BEV rebates at the ZIP code level. We use this analysis to assess distributive justice in the distribution of benefits of government incentives. Finally, we look at both BEV adoption, and the allocation of BEV rebates in neighborhoods in formerly redlined cities in the state to understand how participation and benefits relate to the social and historical context.

#### BEV adoption

To assess equity in BEV adoption, we compare BEV penetration (percent of the light-duty vehicle fleet comprised of BEVs) in 2020 in ZIP codes with different racial and ethnic majorities. We first compute BEV penetration as shown in Equation 1, where *P*_*BEV,z*_ is the BEV penetration in a given ZIP code *z*, *n*_*BEV,z*_ is the number of BEVs in the ZIP code, and *n*_*v,z*_ is the number of vehicles of each vehicle type *v* (e.g., gasoline, diesel, BEV) in the ZIP code.

(Equation 1) $$P_{BEV,z}=\frac{n_{BEV,z}}{\sum n_{v,z}}$$

We merge the ZIP code-level BEV penetration data with demographic data containing the population percentage by race and ethnicity and the median household income in each ZIP code. We group the ZIP codes by racial/ethnic majority (e.g., greater than 50% White) and classify them as high or low income, where high-income is defined as a ZIP code with a median household income greater than or equal to the median income across all ZIP codes in the dataset, and low-income is defined as a ZIP code with a median household income less than the median income across all ZIP codes in the dataset. We then create boxplots showing the median and inner quartile range of the BEV penetration in ZIP codes in each group in 2020. We use racial/ethnic-majority ZIP codes to identify communities with a strong presence of each race/ethnicity. This allows us to understand how BEV adoption rates vary in communities of different races and ethnicities. We also conduct a time series analysis by tracking the mean BEV penetration in ZIP codes with each racial/ethnic-majority and income level from 2010 to 2020, calculating the mean penetration in each category in each year.

#### BEV rebates

To assess equity in the distribution of benefits of government incentives for electric vehicles, we analyze data from the Clean Vehicle Rebate Project,^71^ a statewide incentive program in California. This dataset contains individual records of rebates granted since the beginning of the program as well as ZIP code and census tract information for each entry. We perform this analysis at a ZIP code level to allow comparison with the BEV penetration analysis. Given that census tracts are more granular than ZIP codes, we also perform the analysis at a census tract level, as shown in Figure S15. Results are very similar using both geographical units, with the main distinction being that in the ZIP code analysis, low-income Black-majority ZIP codes have higher rebates per capita than low-income White-majority ZIP codes, whereas in the census tract analysis, the reverse is true. The sample size for low-income Black-majority census tracts is considerably larger (n = 49) than for low-income Black-majority ZIP codes (n = 3). We consider only rebates awarded to individuals for BEVs.

To allow for comparison across ZIP codes of different sizes, we normalize the rebates received in each ZIP code by the population, as shown in Equation 2, where *r*_*z*_ is the rebates per capita in a given ZIP code *z*, *R*_*z*_ is the total number of rebates received in the ZIP code, and *P*_*z*_ is the population in the ZIP code. We merge the rebate data with demographic data at the ZIP code level and compare this metric in racial/ethnic majority ZIP codes with high and low income levels as described in the previous section. Similarly, we track mean rebates per capita over the course of the program’s lifetime.

(Equation 2) $$r_{z}=\frac{R_{z}}{P_{z}}$$

#### BEV adoption and rebates in redlined cities

We use redlining data to identify areas of chronic disinvestment and assess the level of BEV penetration and rebate allocation in these areas. Once we have determined BEV penetration at the ZIP code level, and rebates per capita received at the census tract level, we spatially join these datasets with shapefiles showing the HOLC scores of neighborhoods in historically redlined cities in California^73^. While redlining was a national practice, it was applied only in certain cities. In California, it occurred in eight cities: Fresno, San Diego, San Francisco, San Jose, Stockton, Sacramento, Oakland, and Los Angeles. For this section of the analysis, we include only the data that overlaps with redlined neighborhood boundaries in each city.

We calculate the BEV penetration and rebates per capita in each neighborhood using an area-weighted average of the overlapping ZIP codes and census tracts, as shown in Equations 3 and 4, where *P*_*BEV*__*,*__*n*_ is the percentage of BEV vehicles in neighborhood *n*, *P*_*BEV,z*_ is the percentage of BEV vehicles in ZIP code *z*, *a*_*n,z*_ is the area of the intersection of neighborhood *n* and census tract *z*, *r*_*n*_ is the rebates per capita in neighborhood *n*, *a*_*n*_ is the area of the neighborhood, *a*_*n,t*_ is the area of the intersection of neighborhood *n* and census tract *t*, and *r*_*t*_ is the rebates per capita in census tract *t*. Finally, we group the neighborhoods by HOLC score, and create boxplots showing how the distribution of total BEV rebates per capita and BEV penetration in 2020 compare in neighborhoods with different scores.

(Equation 3) $$P_{BEV,n}=\frac{1}{a_{n}}\sum_{t}a_{n,z}P_{BEV,z}$$

(Equation 4) $$r_{n}=\frac{1}{a_{n}}\sum_{t}a_{n,t}r_{t}$$

#### Sensitivity analysis

##### Census tract analysis

Since rebate data are available at both the census tract and ZIP code level, we conduct the same analysis as discussed in the main text at the census tract level. The results are largely comparable across the two analyses. Figure S15 shows the allocation of rebates per capita in low- and high-income ZIP codes with different racial/ethnic majorities and the evolution of rebate allocation over time.

##### Income cutoff

To assess the sensitivity of the results to our choice of income cutoff, we repeat our analysis of BEV penetration and rebate allocation using a county-specific income cutoff from the California Department of Housing and Community Development to define low income, as shown in Figures S16 and S17.

##### Secondary racial analysis

To account for differing racial percentages across ZIP codes with different racial majorities, we conduct a secondary analysis to understand racial disparities in BEV penetration and rebate allocation. In this analysis, we calculate the population-weighted average BEV penetration and rebates per capita across all ZIP codes in the dataset for each race as shown in Equations 5 and 6, where *P*_*BEV,r*_ is the population-weighted BEV penetration for race *r*, *P*_*BEV,z*_ is the BEV penetration in ZIP code *z*, *n*_*r,z*_ is the population of race *r* in ZIP code *z*, *r*_*r*_ is the rebates per capita for race *r*, and *r*_*z*_ is the rebates per capita in ZIP code *z*.

(Equation 5) $$P_{BEV,r}=\frac{\sum_{z}P_{BEV,z}n_{r,z}}{\sum_{z}n_{r,z}}$$

(Equation 6) $$r_{r}=\frac{\sum_{z}r_{z}n_{r,z}}{\sum_{z}n_{r,z}}$$

## Data Availability

All data used to generate the figures in this work are publicly available and are described in the key resources table. The code used to generate figures is available on Zenodo: https://doi.org/10.5281/zenodo.7865744.
