# Supplementary material for: Assessing justice in California’s transition to electric vehicles
Source: iScience. 2023 May 12;26(7):106856. doi: 10.1016/j.isci.2023.106856 (PMC10391565; doi:10.1016/j.isci.2023.106856)
Supplement: Document S1. Figures S1–S17 and Tables S1 and S2 [file mmc1.pdf]

iScience, Volume *26*

Supplemental information

Assessing justice in California's transition to electric vehicles

Eleanor M. Hennessy and Sita M. Syal

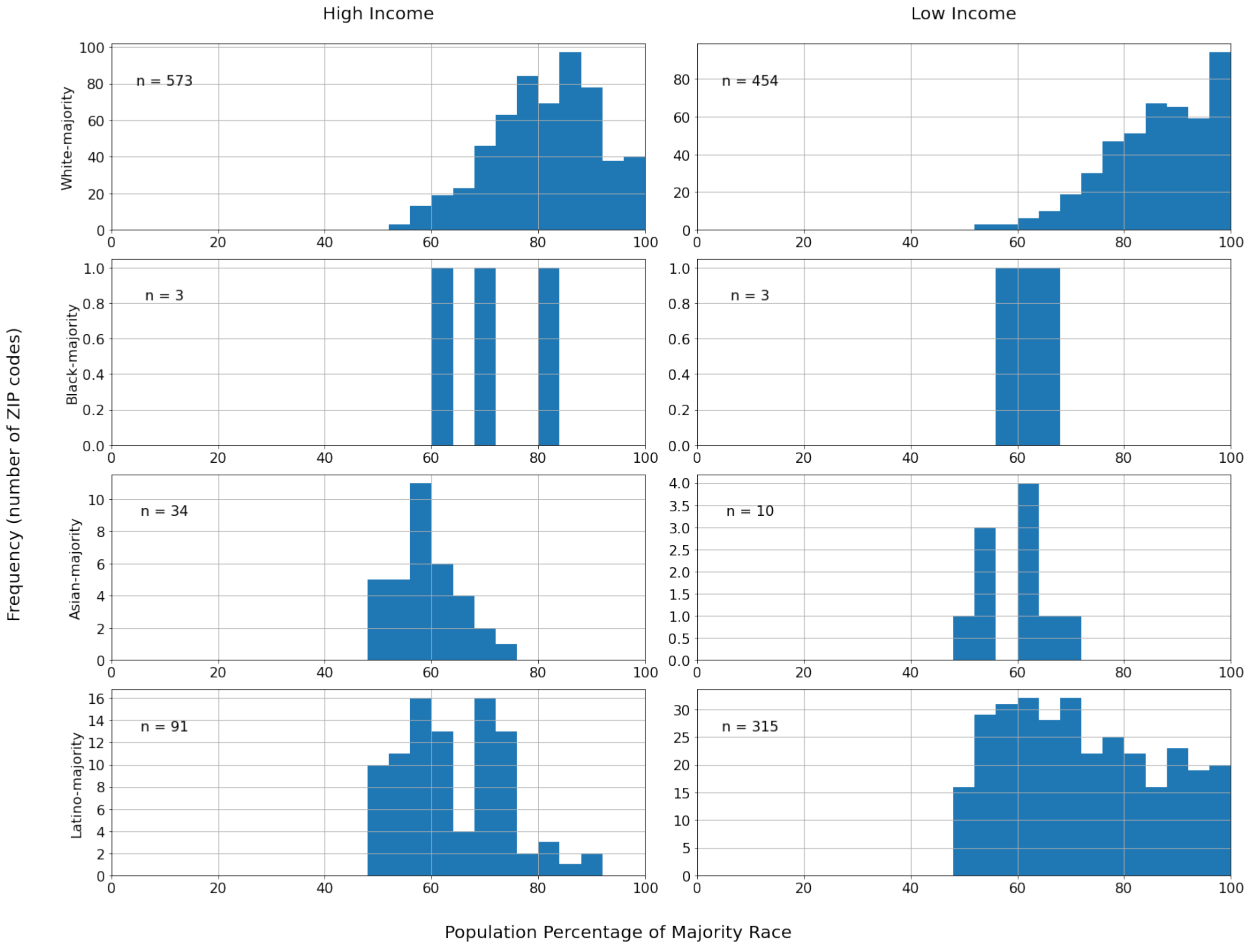

Figure S1: Racial distribution in ZIP codes in each race and income category. Related to Figure 1 and Figure 2.

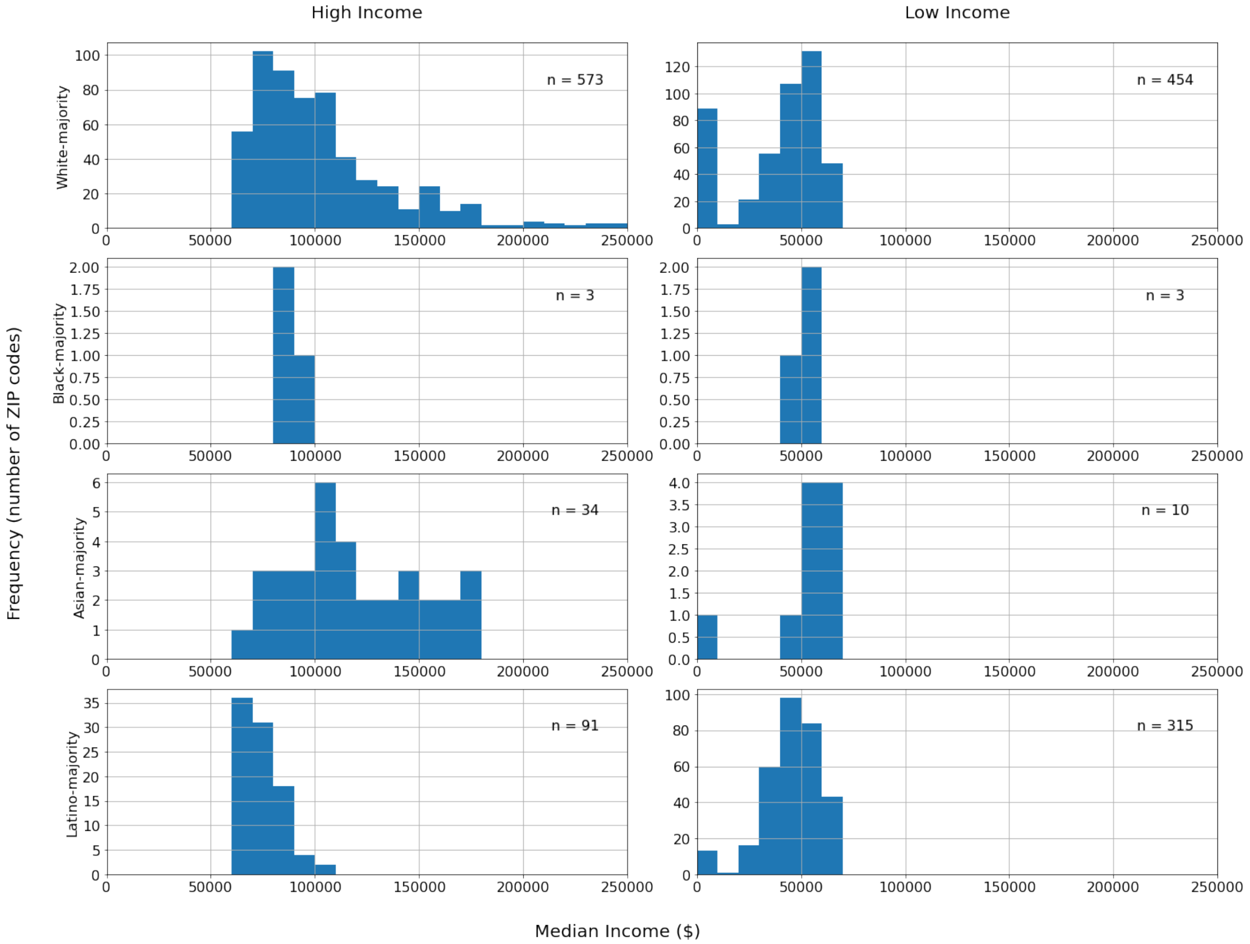

Figure S2: Income distribution in ZIP codes in each race and income category. Related to Figure 1 and Figure 2.

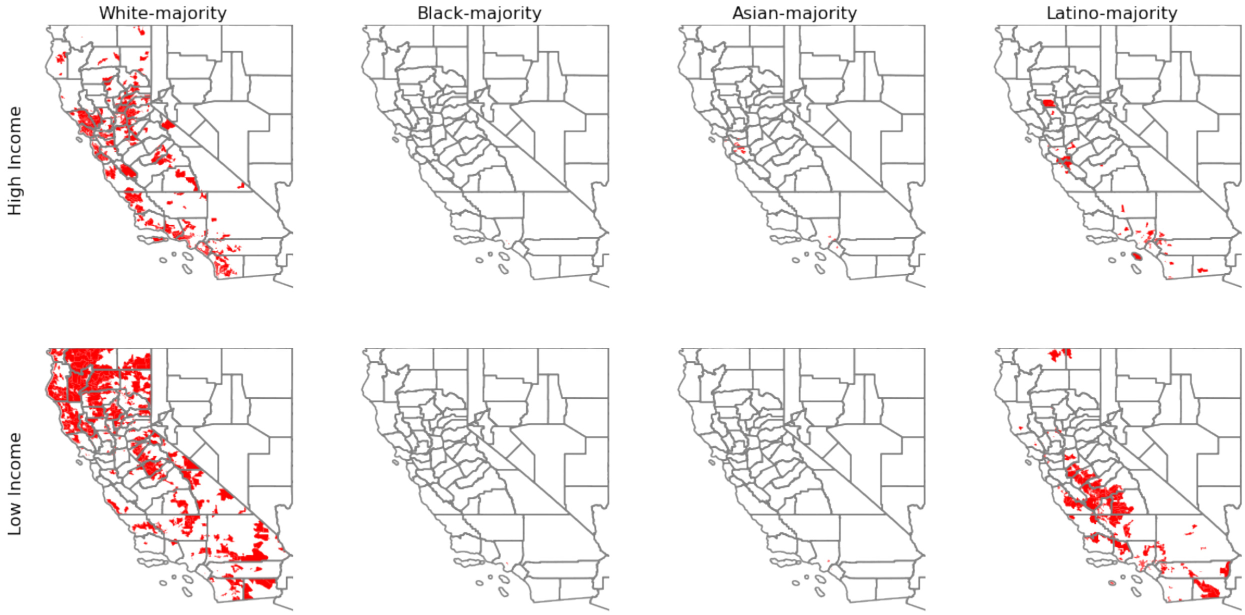

Figure S3: Geographic distribution of ZIP codes in each race and income category. Related to Figure 1 and Figure 2.

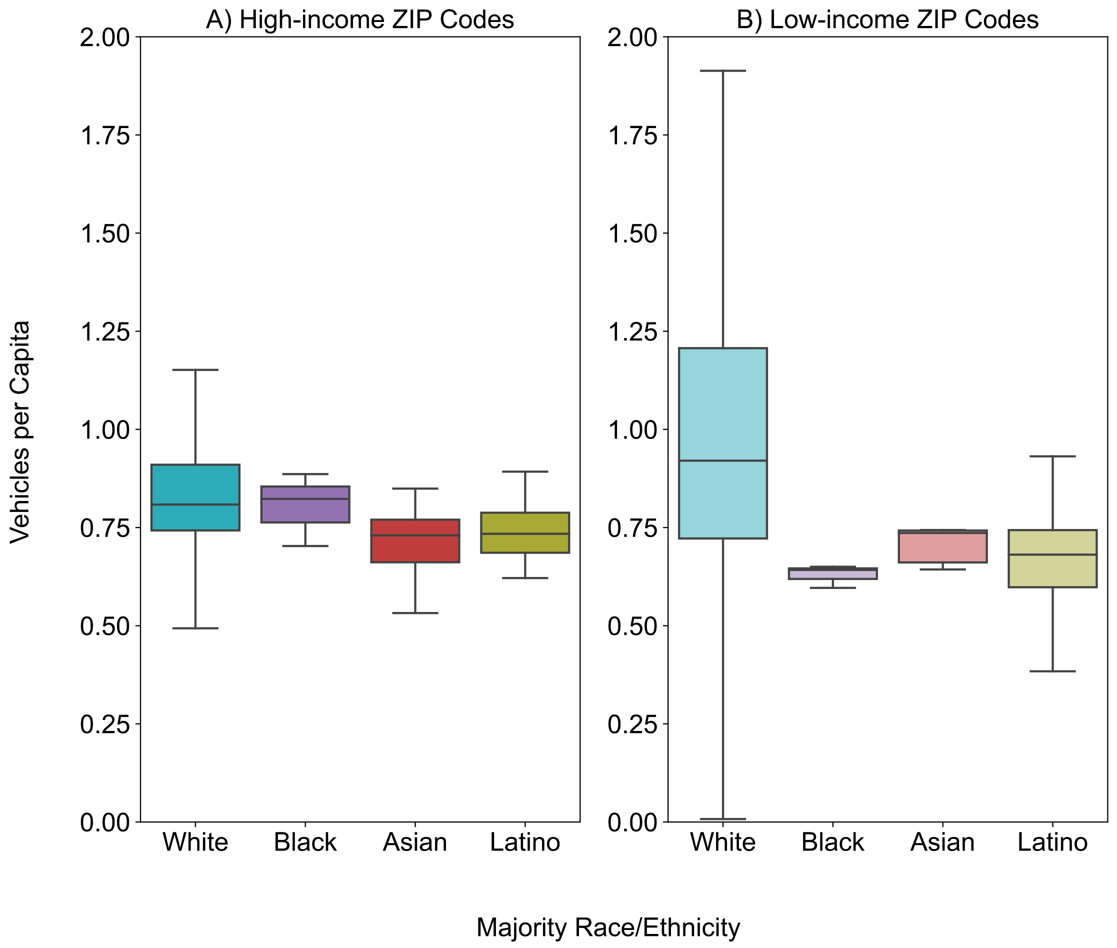

Figure S4: Vehicles per capita in 2020, calculated as the total number of light-duty vehicles in each ZIP code (not limited to BEVs) divided by the total population in each ZIP code. The median vehicles per capita is similar in high and low-income ZIP codes, and across races. In high-income ZIP codes, vehicles per capita are similar across all racial-majorities. In low-income ZIP codes, White-majority ZIP codes have higher vehicles per capita than other groups, but this group also has the widest distribution. Related to Figure 2.

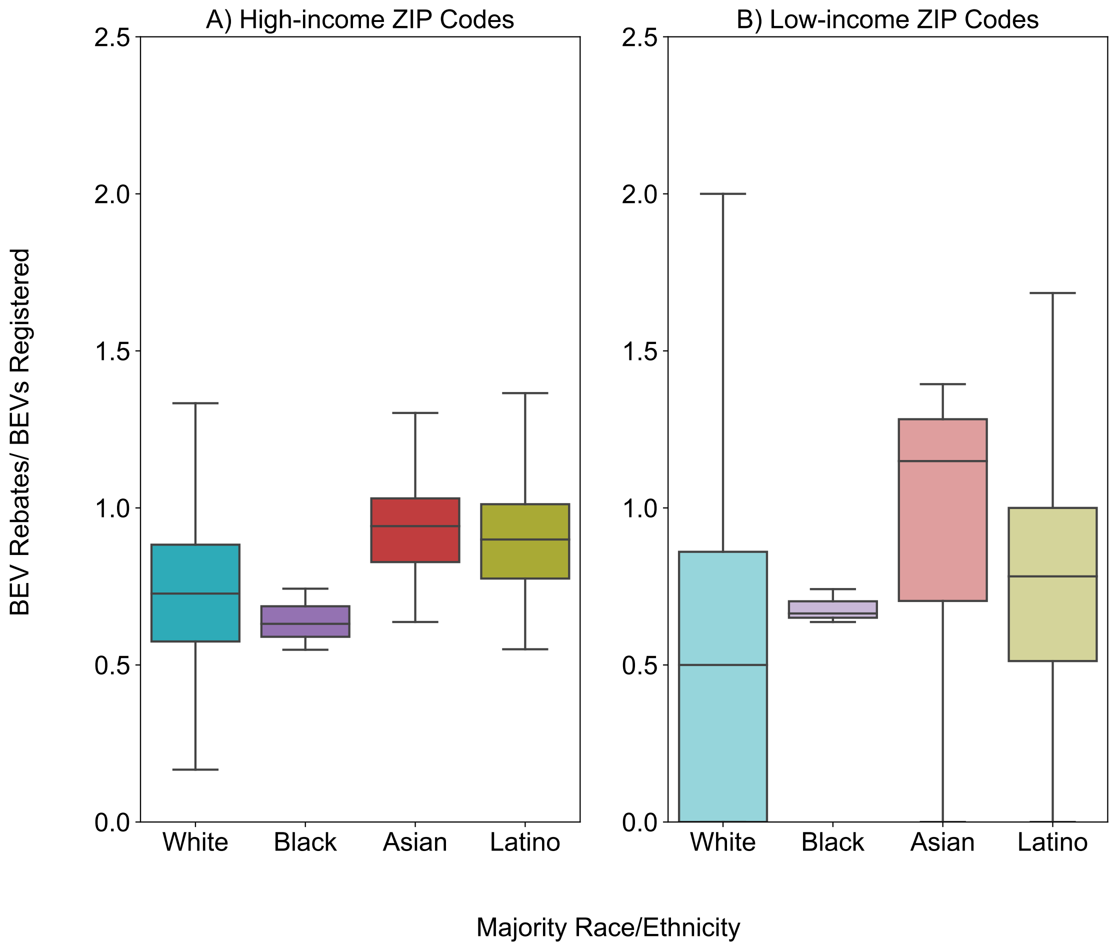

Figure S5: BEV rebates per BEVs registered. Rebates include all BEV rebates received between 2010 and 2020, and BEVs registered includes BEVs registered in 2020. Related to Figure 1 and Figure 2.

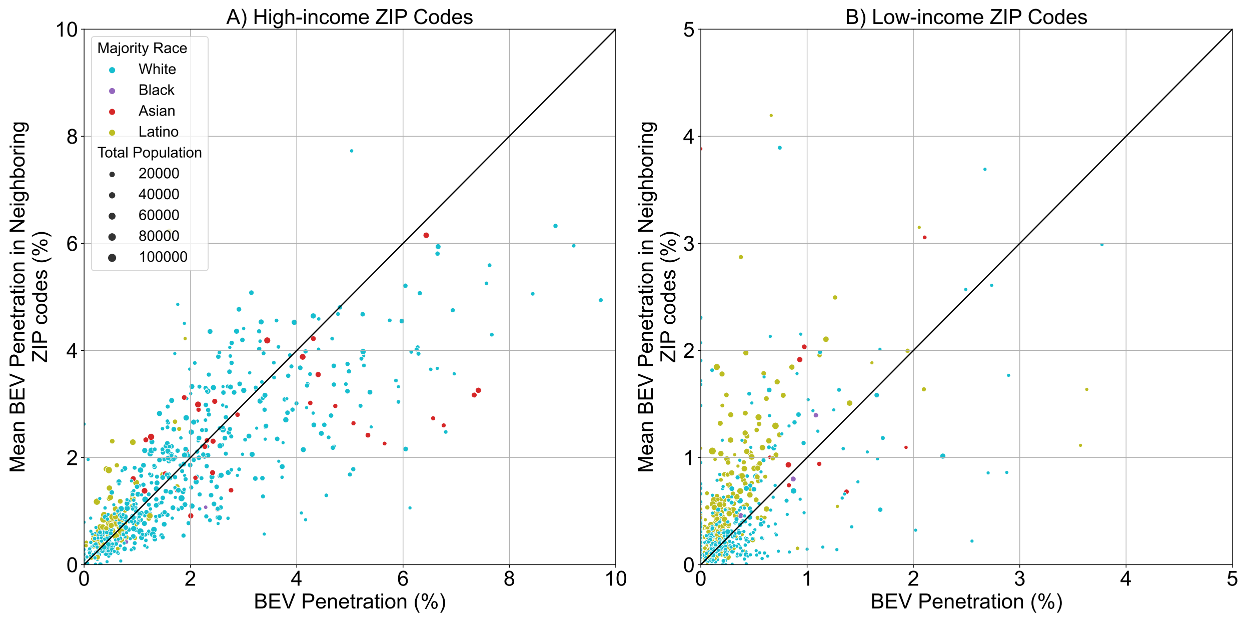

Figure S6: Mean BEV penetration in neighboring ZIP codes vs. BEV penetration in each ZIP code. Related to Figure 1.

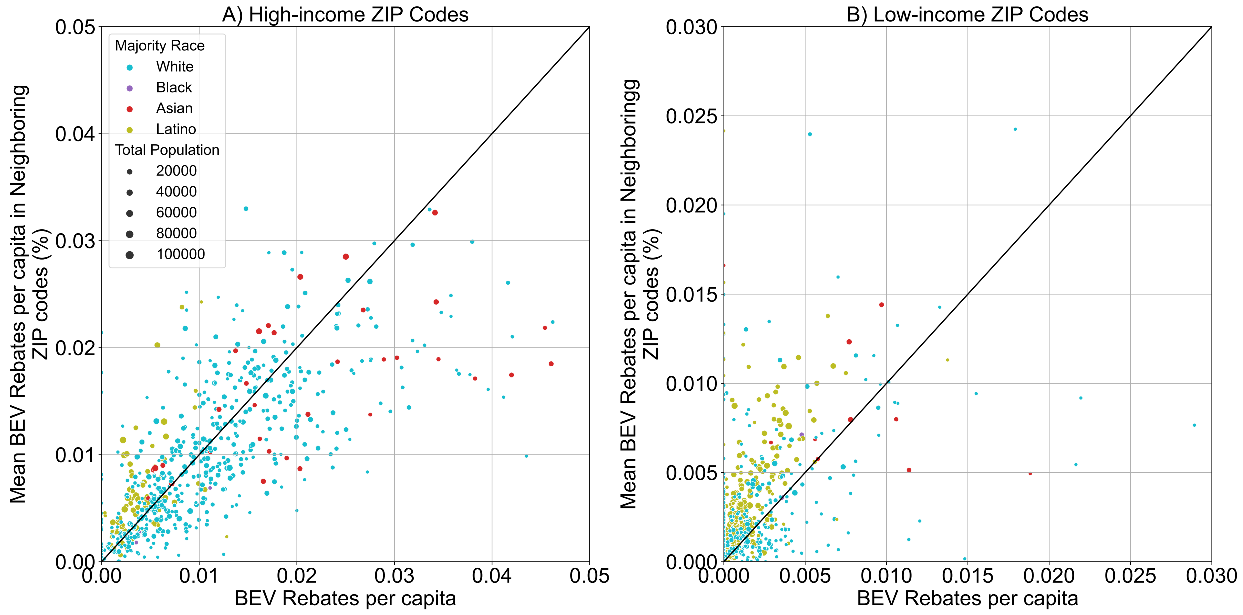

Figure S7: Mean rebates per capita in neighboring ZIP codes vs. rebates per capita in each ZIP code. Related to Figure 2.

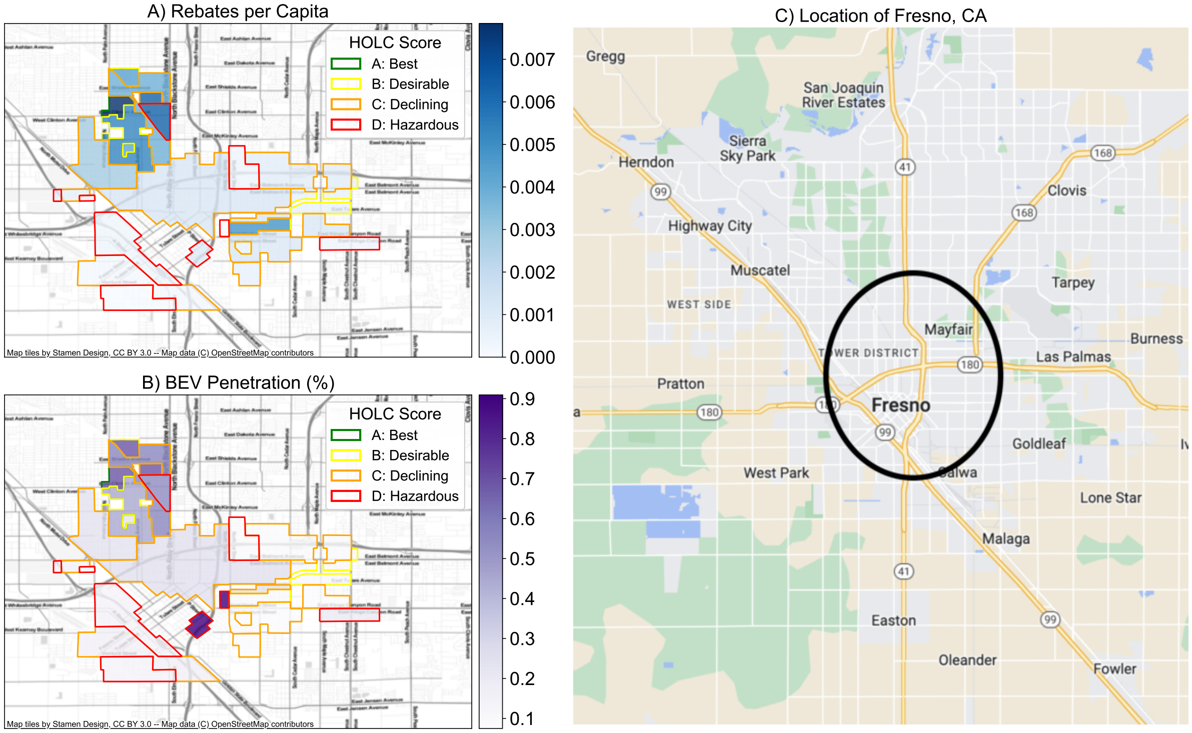

Figure S8: (A) Cumulative rebates per capita awarded in neighborhoods in Fresno, California. Darker blue indicates higher rebates per capita. The color of the outlines corresponds to the HOLC score in the neighborhood, with green corresponding to an “A” grade, yellow corresponding to a “B” grade, orange to a “C” grade, and red to a “D” grade. (B) BEV penetration in 2020 in neighborhoods in Fresno, California. Darker purple indicates higher BEV penetration. The color of the outlines corresponds to the HOLC score in the neighborhood, with green corresponding to an “A” grade, yellow corresponding to a “B” grade, orange to a “C” grade, and red to a “D” grade. (C) Location of Fresno, California. Related to Figure 3.

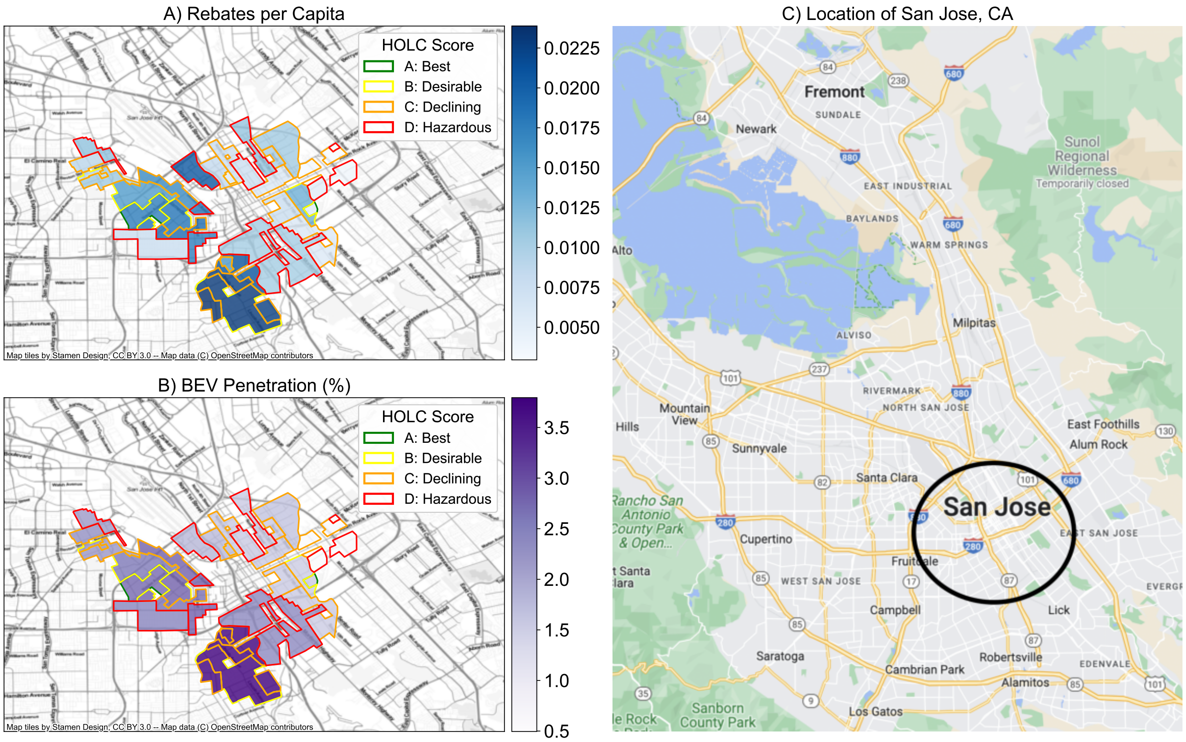

Figure S9: (A) Cumulative rebates per capita awarded in neighborhoods in San Jose, California. Darker blue indicates higher rebates per capita. The color of the outlines corresponds to the HOLC score in the neighborhood, with green corresponding to an “A” grade, yellow corresponding to a “B” grade, orange to a “C” grade, and red to a “D” grade. (B) BEV penetration in 2020 in neighborhoods in San Jose, California. Darker purple indicates higher BEV penetration. The color of the outlines corresponds to the HOLC score in the neighborhood, with green corresponding to an “A” grade, yellow corresponding to a “B” grade, orange to a “C” grade, and red to a “D” grade. (C) Location of San Jose, California. Related to Figure 3.

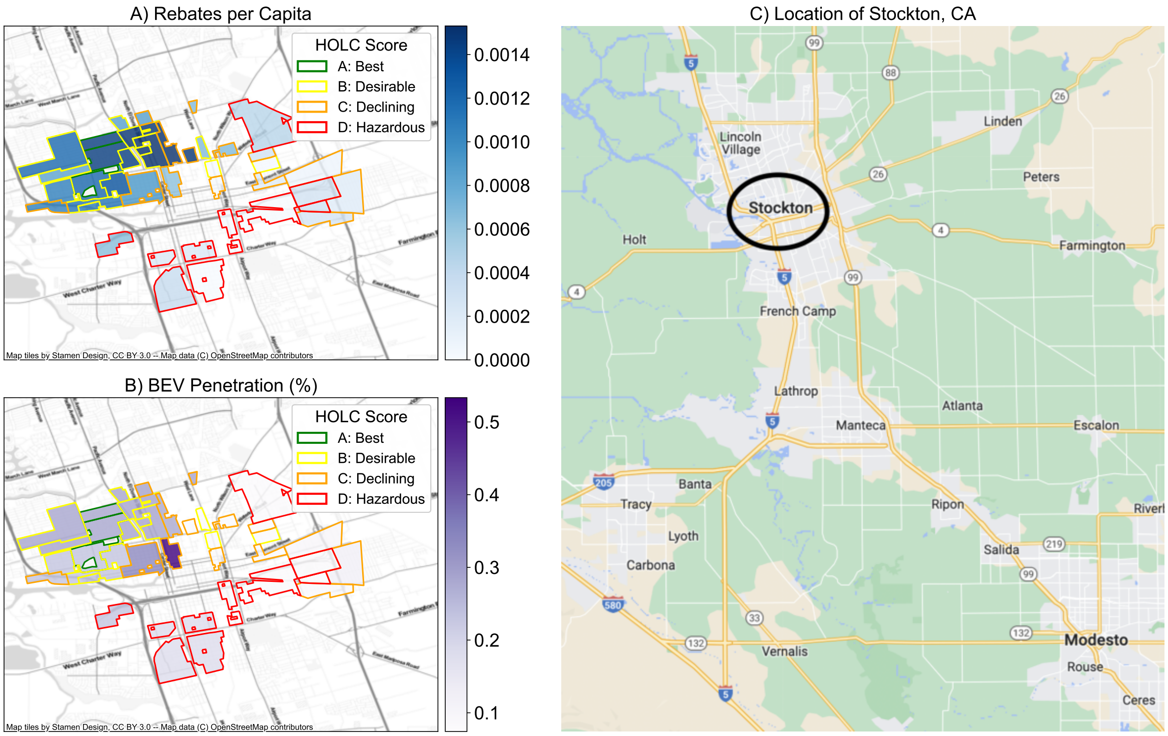

Figure S10: (A) Cumulative rebates per capita awarded in neighborhoods in Stockton, California. Darker blue indicates higher rebates per capita. The color of the outlines corresponds to the HOLC score in the neighborhood, with green corresponding to an "A" grade, yellow corresponding to a "B" grade, orange to a "C" grade, and red to a "D" grade. (B) BEV penetration in 2020 in neighborhoods in Stockton, California. Darker purple indicates higher BEV penetration. The color of the outlines corresponds to the HOLC score in the neighborhood, with green corresponding to an "A" grade, yellow corresponding to a "B" grade, orange to a "C" grade, and red to a "D" grade. (C) Location of Stockton, California. Related to Figure 3.

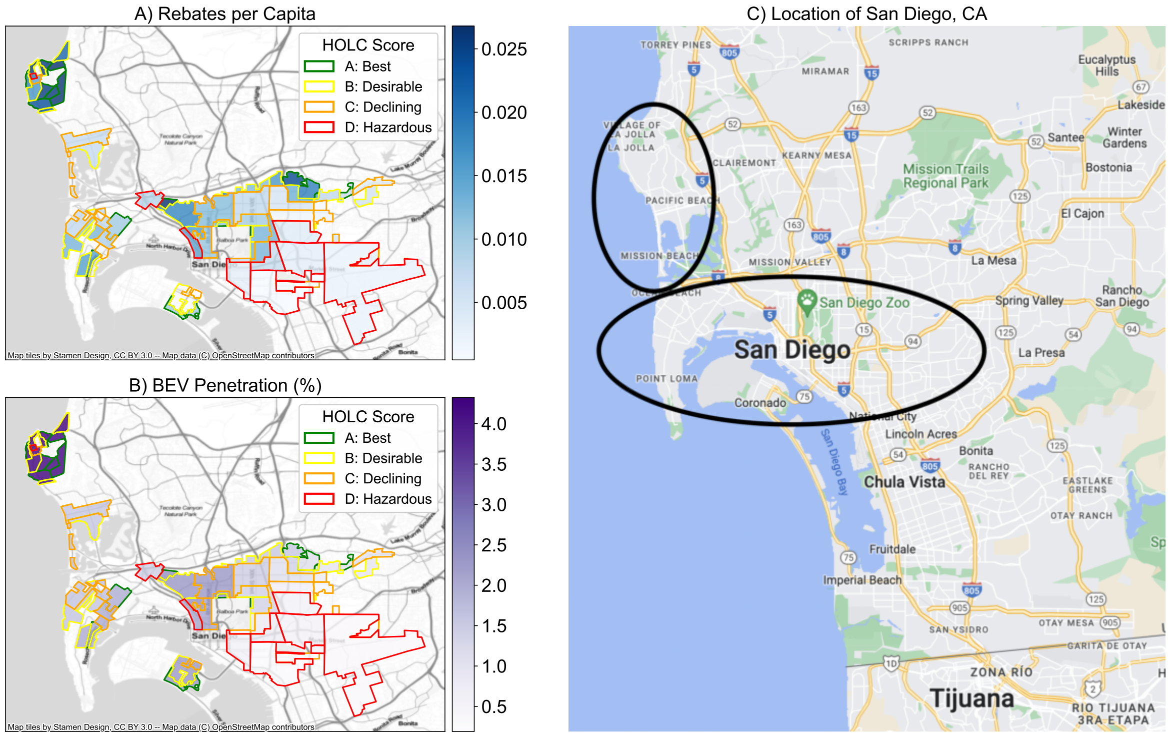

Figure S11: (A) Cumulative rebates per capita awarded in neighborhoods in San Diego, California. Darker blue indicates higher rebates per capita. The color of the outlines corresponds to the HOLC score in the neighborhood, with green corresponding to an “A” grade, yellow corresponding to a “B” grade, orange to a “C” grade, and red to a “D” grade. (B) BEV penetration in 2020 in neighborhoods in San Diego, California. Darker purple indicates higher BEV penetration. The color of the outlines corresponds to the HOLC score in the neighborhood, with green corresponding to an “A” grade, yellow corresponding to a “B” grade, orange to a “C” grade, and red to a “D” grade. (C) Location of San Diego, California. Related to Figure 3.

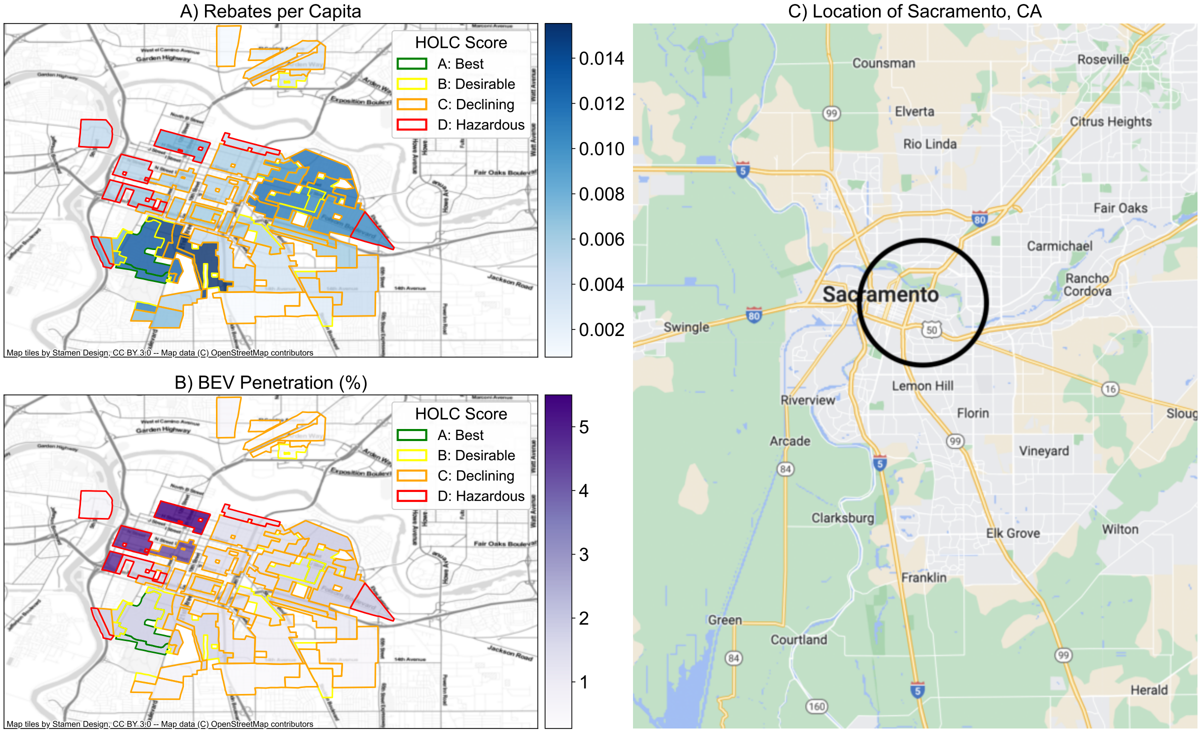

Figure S12: (A) Cumulative rebates per capita awarded in neighborhoods in Sacramento, California. Darker blue indicates higher rebates per capita. The color of the outlines corresponds to the HOLC score in the neighborhood, with green corresponding to an “A” grade, yellow corresponding to a “B” grade, orange to a “C” grade, and red to a “D” grade. (B) BEV penetration in 2020 in neighborhoods in Sacramento, California. Darker purple indicates higher BEV penetration. The color of the outlines corresponds to the HOLC score in the neighborhood, with green corresponding to an “A” grade, yellow corresponding to a “B” grade, orange to a “C” grade, and red to a “D” grade. (C) Location of Sacramento, California. Related to Figure 3.

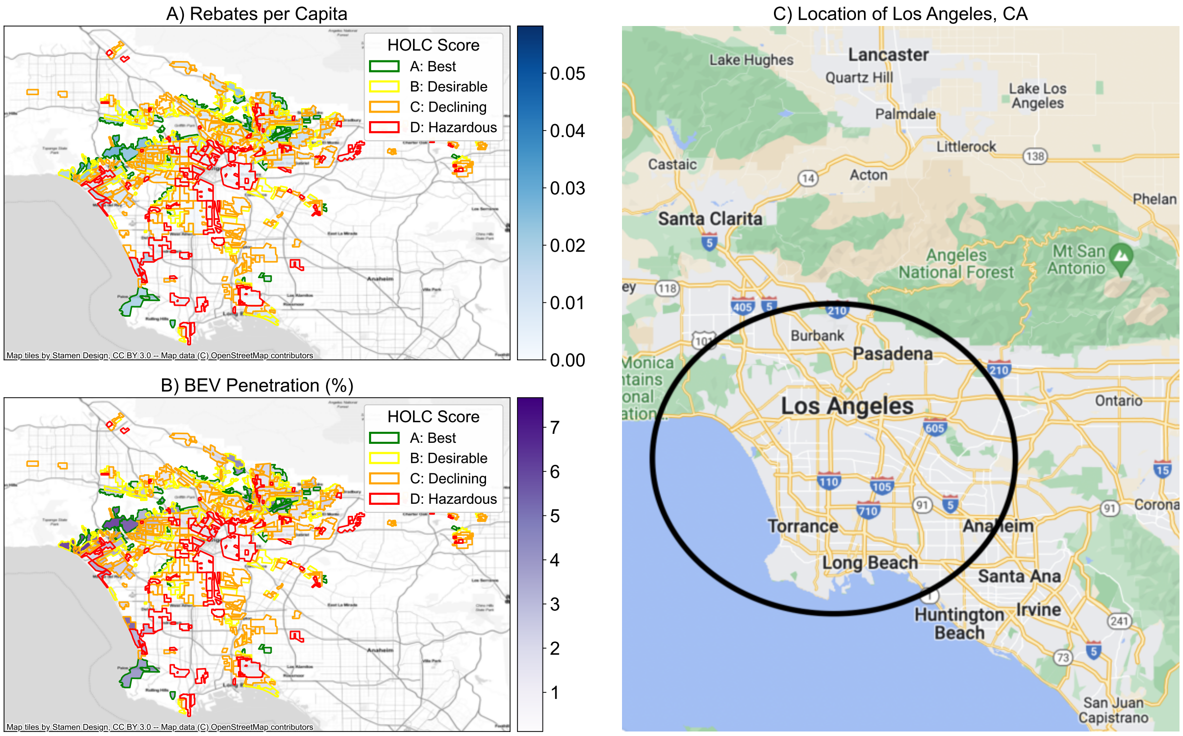

Figure S13: (A) Cumulative rebates per capita awarded in neighborhoods in Los Angeles, California. Darker blue indicates higher rebates per capita. The color of the outlines corresponds to the HOLC score in the neighborhood, with green corresponding to an "A" grade, yellow corresponding to a "B" grade, orange to a "C" grade, and red to a "D" grade. (B) BEV penetration in 2020 in neighborhoods in Los Angeles, California. Darker purple indicates higher BEV penetration. The color of the outlines corresponds to the HOLC score in the neighborhood, with green corresponding to an "A" grade, yellow corresponding to a "B" grade, orange to a "C" grade, and red to a "D" grade. (C) Location of Los Angeles, California. Related to Figure 3.

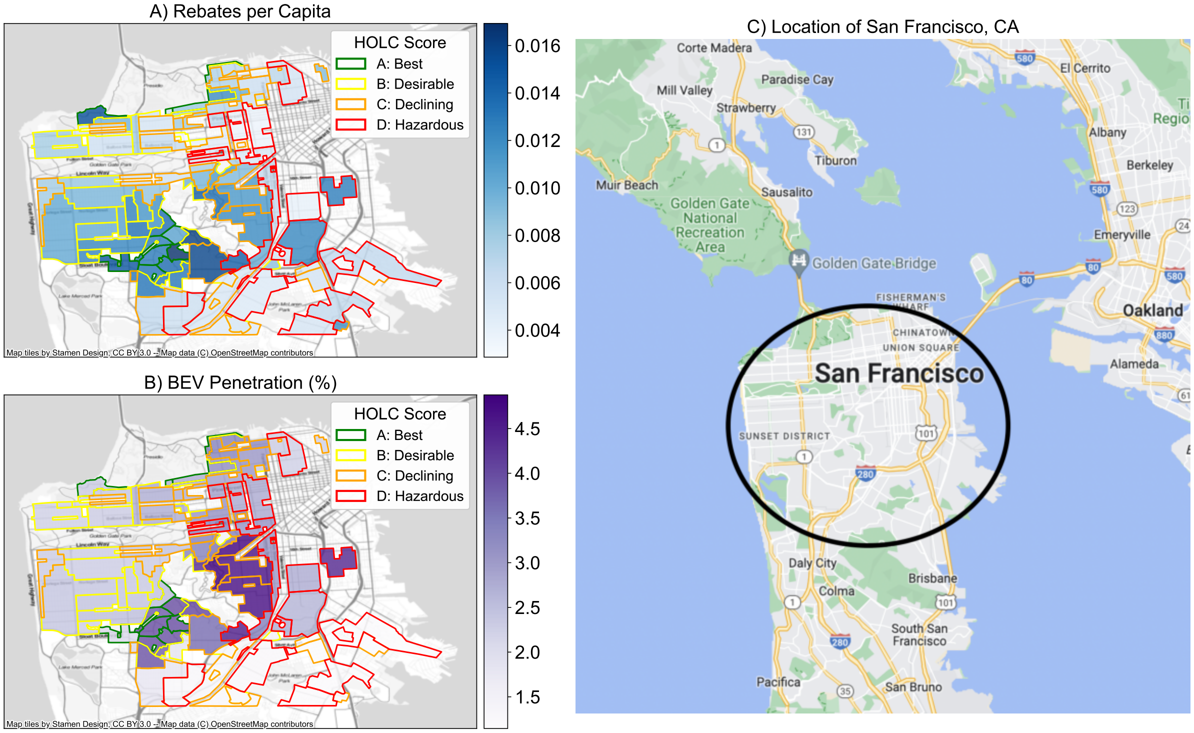

Figure S14: (A) Cumulative rebates per capita awarded in neighborhoods in San Francisco, California. Darker blue indicates higher rebates per capita. The color of the outlines corresponds to the HOLC score in the neighborhood, with green corresponding to an "A" grade, yellow corresponding to a "B" grade, orange to a "C" grade, and red to a "D" grade. (B) BEV penetration in 2020 in neighborhoods in San Francisco, California. Darker purple indicates higher BEV penetration. The color of the outlines corresponds to the HOLC score in the neighborhood, with green corresponding to an "A" grade, yellow corresponding to a "B" grade, orange to a "C" grade, and red to a "D" grade. (C) Location of San Francisco, California. Related to Figure 3.

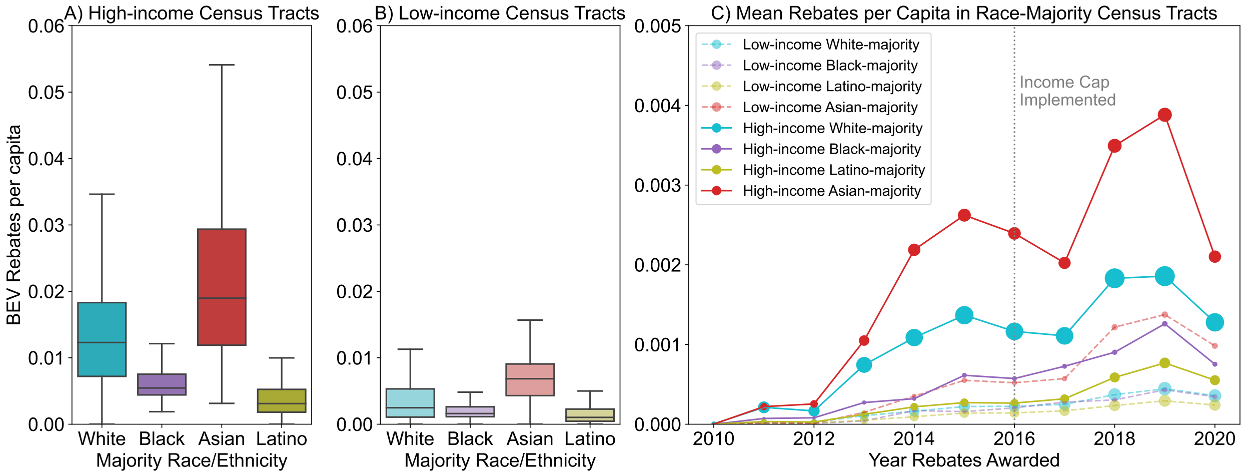

Figure S15: BEV rebates per capita by census tract. Related to Figure 2.

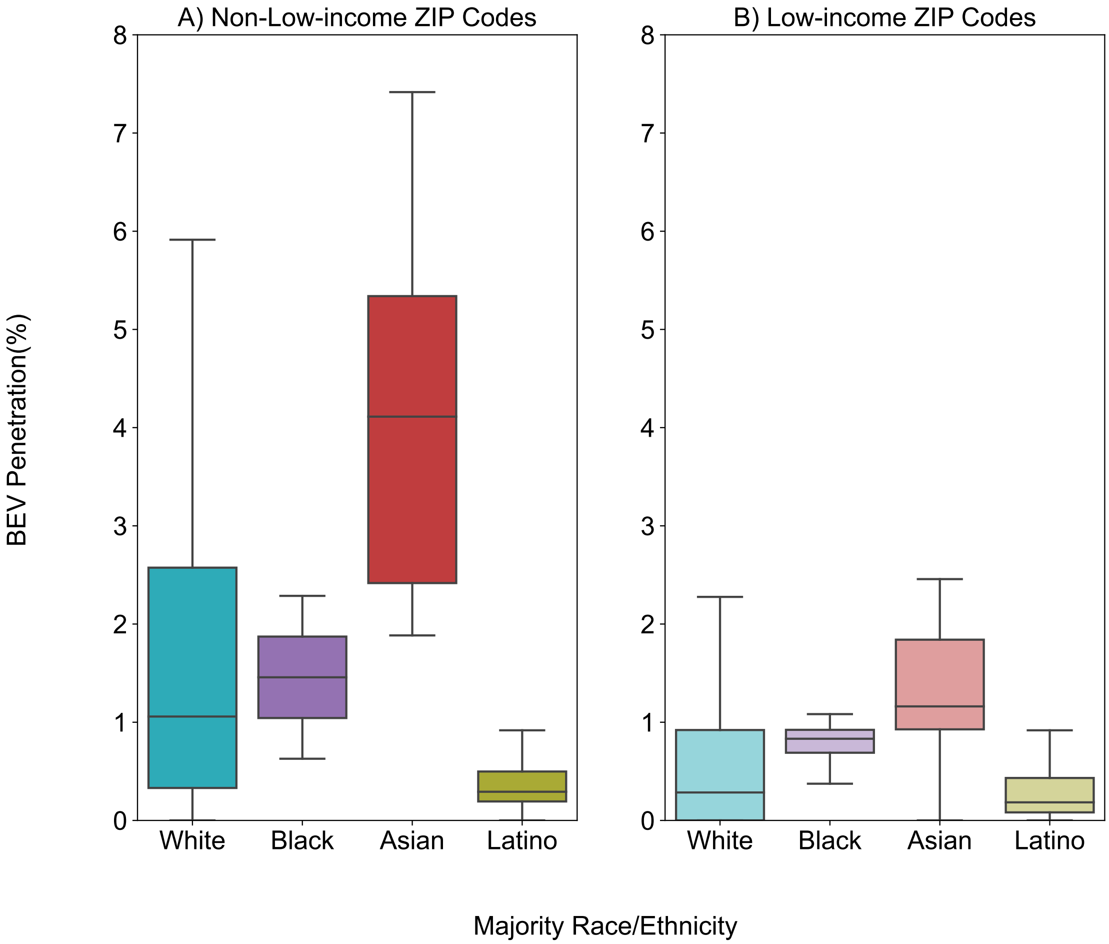

Figure S16: BEV penetration in non-low income and low-income ZIP codes with racial/ethnic majorities. Here low-income is defined on a county basis, using the California Department of Housing and Community Development low-income limit for a 4-person household. Related to Figure 1.

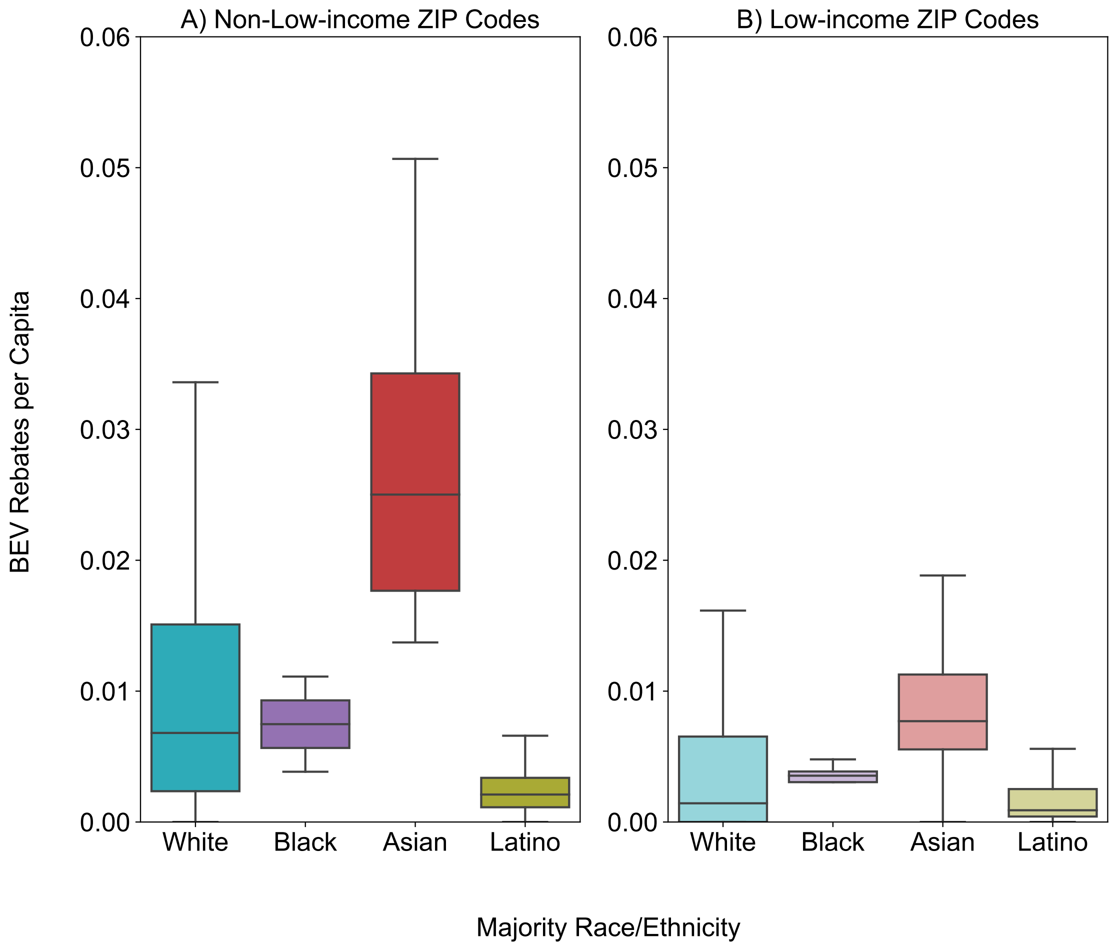

Figure S17: Rebates per capita in non-low Income and low-income ZIP codes with racial/ethnic majorities. Here low-income is defined on a county basis, using the California Department of Housing and Community Development low-income limit for a 4-person household. Related to Figure 2.

	Low-income ZIP codes	High-income ZIP codes
White	0.379	1.79
Black	0.462	1.48
Asian	0.692	2.32
Latino	0.330	1.22

Table S1: Mean BEV percentage by race/ethnicity in high- and low-income ZIP codes, assuming that BEVs are distributed within each ZIP code proportionately to the racial distribution in the ZIP code. Related to Figure 1.

	Low-income ZIP codes	High-income ZIP codes
White	0.00203	0.0102
Black	0.00215	0.00876
Asian	0.00373	0.0141
Latino	0.00185	0.00754

Table S2: Mean BEV rebates per capita by race/ethnicity in high- and low-income ZIP codes, assuming that rebates are distributed within each ZIP code proportionately to the racial distribution in the ZIP code. Related to Figure 2.
